# Diisopropylphenyl-imidazole (DII): A new compound that exerts anthelmintic activity through novel molecular mechanisms

**DOI:** 10.1371/journal.pntd.0007021

**Published:** 2018-12-17

**Authors:** María Gabriela Blanco, María Soledad Vela Gurovic, Gustavo Fabián Silbestri, Andrés Garelli, Sebastián Giunti, Diego Rayes, María José De Rosa

**Affiliations:** 1 Instituto de Investigaciones Bioquímicas de Bahía Blanca (INIBIBB) CCT UNS-CONICET, Bahía Blanca, Argentina; 2 Dpto de Biología, Bioquímica y Farmacia, Universidad Nacional del Sur, Bahía Blanca, Argentina; 3 CERZOS UNS-CONICET CCT, Bahía Blanca, Argentina; 4 Dpto de Química, Universidad Nacional del Sur (UNS)-CONICET, Instituto de Química del Sur (INQUISUR), Bahía Blanca, Argentina; University of Georgia, UNITED STATES

## Abstract

Nematode parasites cause substantial morbidity to billions of people and considerable losses in livestock and food crops. The repertoire of effective anthelmintic compounds for treating these parasitoses is very limited, as drug development has been delayed for decades. Moreover, resistance has become a global concern in livestock parasites and is an emerging issue for human helminthiasis. Therefore, anthelmintics with novel mechanisms of action are urgently needed. Taking advantage of *Caenorhabditis elegans* as an established model system, we here screened the nematicidal potential of novel imidazolium and imidazole derivatives. One of these derivatives, diisopropylphenyl-imidazole (DII), is lethal to *C*. *elegans* at both mature and immature stages. This lethal effect appears to be specific because DII concentrations which prove to be toxic to *C*. *elegans* do not induce significant lethality on bacteria, *Drosophila melanogaster*, and HEK-293 cells. Our analysis of DII action on *C*. *elegans* mutant strains determined that, in the adult stage, null mutants of *unc-29* are resistant to the drug. Muscle expression of this gene completely restores DII sensitivity. UNC-29 has been largely reported as an essential constituent of the levamisole-sensitive muscle nicotinic receptor (L-AChR). Nevertheless, null mutants in *unc-63* and *lev-8* (essential and non-essential subunits of L-AChRs, respectively) are as sensitive to DII as the wild-type strain. Therefore, our results suggest that DII effects on adult nematodes rely on a previously unidentified UNC-29-containing muscle AChR, different from the classical L-AChR. Interestingly, DII targets appear to be different between larvae and adults, as *unc-29* null mutant larvae are sensitive to the drug. The existence of more than one target could delay resistance development. Its lethality on *C*. *elegans*, its harmlessness in non-nematode species and its novel and dual mechanism of action make DII a promising candidate compound for anthelmintic therapy.

## Author summary

Intestinal helminth infections affect approximately one-third of the world’s population, particularly in developing countries. Paradoxically, drug development in this area has been delayed for years. In addition, resistance to currently available drugs is also an emerging global concern. Therefore, there is an urgent need for new and effective anthelmintics. In this work, we used *C*. *elegans* as a model for parasitic nematodes to screen the anthelmintic activity of several imidazole-derivative compounds. We found a compound, diisopropylphenyl-imidazole (DII), that is lethal to both mature and immature stages of *C*. *elegans*. The DII nematicidal mechanism of action depends on a novel UNC-29-containing AChR in adult *C*. *elegans* muscle. Since this mechanism is different from those of currently used anthelmintics, it could constitute a therapeutic option when traditional anthelmintic agents fail. In addition, we found that the DII larvicidal effect depends on a different target to that of adult stages. The fact that DII produces lethality through different targets may delay resistance development. The specificity and novel mode of action of DII, which includes differential targeting in larvae and adult nematodes, support its potential as a promising drug candidate to treat helminthiasis.

## Introduction

Parasitic nematodes can infect humans, companion animals, livestock and crops producing a devastating impact on human life quality and economy. The World Health Organization (WHO) estimates that 25–30% of the human world population is infected with at least one parasite (http://www.who.int/intestinal_worms/more/en/). This prevalence is even higher in some developing countries. Human helminthiasis consequences are particularly serious in children, impairing growth, nutrition, cognition, and school performance. Although helminth parasites are a significant public health and economic concern, the development of novel pharmacological agents to treat helminthiasis has been delayed for decades and the repertoire of available anthelmintics is limited [1, 2]. The fact that only one drug, tribendimidine, was developed and entered into human clinical trials in the last 30 years [3, 4] is clear evidence of this lack of interest in developing new anthelmintics. On the basis of their mode of action, anthelmintic drugs can be grouped into two main classes: those that exert rapid effects acting through membrane ion channels and those that affect biochemical processes and act more slowly [1, 5, 6]. Since drug-resistant parasitic nematodes have been reported for all classes of currently used anthelmintics [7–10], there is an urgent need to advance in pharmacological research to develop new antiparasitic drugs.

To develop new bioactive molecules, the pharmaceutical industry has historically centered its attention on heterocyclic compounds [11]. Due to their capacity to bind to several biological targets, nitrogen-containing heterocycles such as imidazole rings, have been largely used as powerful scaffolds for the development of bioactive molecules [12]. A wide spectrum of activities, including analgesic, anti-inflammatory, antiviral, anticonvulsant, insecticidal, antifungal, and antiparasitic actions have been described for imidazole-containing compounds [13]. The fact that the imidazole aromatic heterocycle is an important building block of several essential biomolecules probably underlies the biological effects of these compounds [14]. Many of the currently used anthelmintics are imidazole-derivatives [15, 16]. The mechanisms underlying the antiparasitic effect of these imidazole-derivatives are diverse. For instance, levamisole causes nematode spastic paralysis through the potent activation of a muscle nicotinic receptor (AChR) [17, 18], whereas the anthelmintic action of benzimidazoles (e.g albendazole and mebendazole) arises from their capacity to inhibit the worm′s tubulin polymerization [19]. Therefore, while the presence of the imidazole ring appears to be important for bioactivity, it does not restrict the molecular targets where imidazole-containing anthelmintics can act.

Due to their complex life cycles, growing and maintaining parasitic nematodes in standard laboratory conditions is challenging. Moreover, the limited repertoire of genetic engineering techniques available for these nematodes hampers the study of molecular mechanisms [20]. In this context, the use of the non-parasitic nematode *C*. *elegans* has emerged as a model for parasitic roundworms [16, 21, 22]. *C*. *elegans* is a free-living nematode that shares phylum-specific properties with parasitic roundworms and has been extensively used as an inexpensive, safe and powerful model in biomedical research [23, 24]. Forward genetic techniques in *C*. *elegans* have played a pivotal role in the understanding of mechanisms of action and resistance of most known anthelmintic agents [19, 25–29]. Recent reports have shown that those compounds that induce *C*. *elegans* death are very likely to also kill parasitic nematodes [30], highlighting the potential of *C*. *elegans* as an anthelmintic screening platform for drug discovery.

In this work, we synthesized imidazolium and imidazole derivatives and screened their anthelmintic potential on *C*. *elegans*. One of these derivatives, diisopropylphenyl-imidazole (DII), is lethal to *C*. *elegans* at both mature and immature stages. We found that DII action on adult nematodes relies on a previously unidentified UNC-29-containing muscle AChRs. Strikingly, in larvae, DII lethality does not depend on UNC-29. The fact that DII target is different between larvae and adults, could be important for delaying nematode resistance acquisition. In contrast to its effects on *C*. *elegans*, the DII lethality on the non-nematode species tested appears to be significantly reduced. Its specificity and its novel mode of action, that includes differential targeting in larva and adult nematodes, support the potential of DII as a promising drug candidate to treat helminthiasis.

## Materials and methods

### Synthesis

#### General procedures

All reactions were carried out under a dry nitrogen atmosphere by using Schlenk techniques. Organic solvents were dried and distilled under nitrogen and degassed prior to use. Unless otherwise stated, reagents were obtained from commercial sources and used as received. ^1^H- and ^13^C-NMR spectra were recorded on a Bruker ARX 300 (300.1 MHz for ^1^H, 75.5 MHz for ^13^C) using D_2_O or CDCl_3_ as solvents. ^1^H- and ^13^C-NMR spectra for each compound are shown in S1 Appendix. C, H, and N analyses were performed by The Analytical Services of the Universidad Nacional del Sur (Argentina) with an Exeter Analytical Inc. CE-440 microanalyzer.

#### Sulfonate-imidazolium salts

The sulfonate-imidazolium salts **1**, **2**, **5** and **6** were synthesized in high yields by quaternization of the corresponding *N*-substituted imidazoles with 1,3-propanesultone according to reported methods [31] (S1 Fig). The imidazolium salt **9** was synthesized from 2,6-diisopropylaniline, following the method previously published [32].

#### Imidazolium salts with bromide or chloride as counter ions

Imidazolium salts **3** and **4** were synthesized following the method published by Perry *et al*. [33], salts **7** and **8** were prepared according to procedures reported by Hintermann [34]. The intermediate **10** and **11** were prepared following the Gardiner and co-workers′procedure (S1 Fig) [35]. In particular, the purity of 11 was assessed by elemental analysis which yielded the following results: C, 77.39; H, 8.97; N, 11.07. This is consistent with the expected values for C_15_H_20_N_2_ (Anal. Calcd: C, 78.90; H, 8.83; N, 12.27).

### *Drosophila melanogaster* larval viability

All experiments were done at 25°C, under a 12:12 dark:light cycle using the *w*^*1118*^ strain. Mated females were allowed to lay eggs for 4 h on apple juice egg laying plates supplemented with a thin layer of normal fly food [36]. 2 h after the initiation of egg laying was considered time "0". 30–40 late L2 larvae (66 h after egg laying) were transferred from the plates to tubes containing 3 ml of fly food with 300, 600 and 1200 μM DII or the corresponding concentration of dimethylsulfoxide (DMSO, molecular biology grade, AppliChem). Under these conditions, larvae were exposed to DII until they left the food to pupariate about 40 h later. Larval viability was calculated as the percentage of larvae that reached the pupal stage.

To add drugs, fly food was melted and cooled to 60°C, 3 ml of food were dispensed in tubes and an appropriate volume of DMSO or drug was added and thoroughly mixed. Control tubes without DMSO and sodium azide were prepared following the same procedure. The experiment was independently repeated at least three times.

### Minimum inhibitory (MICs) and bactericidal concentrations (MBCs)

The strains used in this study were *Escherichia coli* ATCC 25922 and *Staphylococcus aureus* ATCC 25923, kindly provided by Dr. Ziegler (Bacillus Genetic Stock Center, The Ohio State University). Stocks were stored in Mueller Hinton Broth (MHB, Biokar Diagnostics, France) supplemented with glycerol 20% at -70°C. Before running each test, the strains were incubated for 48 h at 37°C in MHB. Finally, the bacterial strains were sub-cultured in MHB at 37°C for 24 h.

The solutions of antimicrobial agents and imidazolium salts were prepared following the Clinical and Laboratory Standards Institute (CLSI) guidelines for determination of Minimum Inhibitory Concentrations by the microdilution method (CLSI 2009). Briefly, stock solutions of 10 mg/ml of imidazolium salts were prepared in DMSO, and two-fold dilutions from these stocks were prepared in sterile saline water (0.85% w/v NaCl) to set a dilution range of 0.125 to 254 μg/ml (equivalent to 0.06–1.37 mM and 0.05–1.11 mM for MI and DII, respectively). The maximum percentage of DMSO in the wells was 2.5%. Controls of DMSO 2.5% and ciprofloxacin were included for each strain. Cultures were incubated as described above and adjusted to OD_625nm_ ~ 0.08–0.13 by the direct colony suspension method in saline water and finally adjusted to 2–8 10^5^ CFU/ml by diluting 1:150 in MHB before testing. A volume of 100 μl of each sample dilution in saline water was transferred to the microplate well. Finally, an equal volume of the inoculum was added. The MIC was the lowest concentration at which there was no visible growth when compared with controls after 24 h at 37°C. To assess the minimum bactericidal concentration (MBC), 20 μl aliquots were taken from the wells where no visible growth was observed, seeded on petri dishes and covered with MH agar (MHA) at 45°C. The compounds were considered bactericidal at the concentration at which no visible colony was observed on the plate after 24 h at 37°C. All conditions were run at least in duplicate. The results were obtained from at least 3 independent experiments.

### Human cell toxicity

HEK-293 cells were cultured in DMEM medium (Gibco, USA) containing 10% fetal bovine serum (FBS) (Gibco, USA) and penicillin/streptomycin (Gibco, USA) at 37°C in an atmosphere containing 5% CO_2_. Before DII exposure, cells were dissociated using trypsin-EDTA and seeded in 96 multiwell plates at a density of 1x10^4^ cells/ml. Cells were resuspended in fresh medium and different drug concentrations were subsequently added. After 8 h of incubation with 0, 200 and 600 μM of DII or 96 h of incubation with 0 and 100 μM of DII, cells were washed 3 times with PBS and propidium iodide (PI, final concentration 50 μg/ml) was added to stain dead cells. PI incubation was performed for 10 min in the dark at room temperature. DMSO (0–0.7%) and chloroquine (50–200 μM) were used as vehicle and positive controls, respectively. Images were acquired with a Nikon Eclipse TE 2000 fluorescence microscope. The number of stained cells was analyzed manually. At least 3 independent experiments were analyzed.

### *C*. *elegans* assays

***C*. *elegans* culture and maintenance**: Worms were cultured and maintained on Nematode Growth Medium (NGM) agar plates seeded with *E*. *coli* OP50 as a food source at 20°C [37, 38]. For all experiments, animals were age-synchronized. *C*. *elegans* Bristol strain N2, anthelmintic resistant strains CB3474 *ben-1(e1880)III*, ZZ26 *unc-63(x26)I*, DA1316 *avr-14(ad1305)I; avr-15(vu227)V; glc-1(pk54)V*, CB1072 *unc-29(e1072)I*, CB407 *unc-49(e407)III*, CB904 *unc-38(e264)I*, VC1041 *lev-8(ok1519)X*, RB2355 *lev-1(ok3201)IV*, and RB918 *acr-16(ok789)V* and *E*. *coli* OP50 were provided by the CGC, which is funded by NIH Office of Research Infrastructure Programs (P40 OD010440).

#### Screening imidazole compounds

100–150 young adult wild-type worms were exposed to these compounds (150 μg/ml) in liquid medium (S-Basal) containing *E*. *coli* OP50. Micromolar concentrations for each compound are: 487 μM for **1**, 428 μM for **2**, 410 μM for **3**, 375 μM for **4**, 789 μM for **5**, 735 μM for **6**, 389 μM for **7**, 319 μM for **8**, 263 μM for **9**, 806 μM for **10** and 657 μM for **11**. Compounds were dissolved in water or DMSO according to their solubility. After 72 h, animal survival was analyzed. An animal was considered dead if it made no movement in response to gentle prodding with a worm pick. At least three independent experiments were analyzed.

#### Lethality assays

Dose-dependent lethality was quantified by exposing 40 L4s to DII (0–1000 μM) dissolved in solid medium for 8 and 24 h at 20°C. For survival to 96 h of DII exposure the temperature used was 13°C to avoid food depletion.

To evaluate DII nematicidal effects on mutant strains two different conditions were tested: i) ~40 L4s were exposed to DII (600 μM) in NGM plates and survival was scored at 4, 8, 24, 48 and 72 h, ii) ~40 L4s were exposed to 50 μM for 96 h and survival was subsequently scored. Animals were considered dead if they failed to respond to gentle prodding with the worm pick. At least three independent experiments were analyzed.

#### Recovery assays

To evaluate the recovery capacity of worms treated with DII, ~100 L4 worms were transferred to NGM plates containing DII (600 μM). After 4 and 8 h of exposure, those animals that survived were transferred to regular NGM plates. Worm viability was scored 24 h later. Results are expressed as the percentage of alive animals 24 h after being transferred to regular NGM plates. The experiment was independently repeated three times.

#### Acute paralysis assays

Acute paralysis assays were carried out in microtiter plates containing the drug dissolved in M9 buffer. A final volume of 100 μl per well was used. 30 L4 wild-type worms were transferred to each well and after 10 minutes the number of paralyzed animals (not thrashing) were counted. Each drug concentration was tested in two wells (containing 30 animals each) within a single experiment. We tested a range of concentrations of levamisole and DII (from 1 to 800 μM). The experiment was independently repeated at least three times.

#### Worm cut preparation

Cutworm assays were performed as previously described with slight modifications [26, 39]. Briefly, synchronized young adult animals were mounted in a glass microscope slide containing Artificial Perienteric Fluid (APF) (67 mM NaCl, 67 mM NaCH_3_COOH, 3 mM KCl, 15.7 mM MgCl_2_, 3 mM CaCl_2_ and 5 mM Tris, pH 7.6). 10 animals were severed between the middle and tail, approximately two-thirds from the anterior end. Worms were subsequently transferred to a multi-well plate containing APF or drug-APF and paralysis was evaluated after 10 minutes. All experiments were performed at least three times.

#### Body length measurements

~10 L4s were transferred to NGM plates containing levamisole or DII at 600 μM. Vehicle (0.75% DMSO) was used as a control. After 2 h, images were acquired and body length was measured using FIJI-ImageJ software. At least three independent experiments were analyzed.

#### Egg-laying assays

Age-synchronized gravid worms were transferred to solid NGM plates containing different drug concentrations (100–300 μM). Each concentration was evaluated in triplicate and DMSO was used as a control. After 1 h, the number of laid eggs per worm was counted. This experiment was independently repeated three times.

#### Toxicity at early developmental stages

Briefly, gravid worms were collected and exposed to an alkaline hypochlorite solution (0.5 M NaOH and 1% NaOCl) to destroy adult worm tissues (eggs are resistant to bleach due to their chitin eggshell). Eggs were subsequently isolated with a sterile 60% sucrose solution and seeded on 35 mm NGM plates containing DII (100, 300 and 600 μM) or DMSO as a control. Hatched animals were quantified after 48 h. The results were expressed as follows: Number of living animals x 100/ Total number of eggs seeded. This experiment was independently repeated three times.

For ovicidal assessment, isolated eggs were seeded in NGM plates containing DII (600 μM) and video-recorded for 12 h. An egg was considered as non-viable if no hatching was observed during the recorded time.

To assess developmental rate, eggs were seeded on NGM plates containing DII (600 μM) and 0.75% DMSO (vehicle). The number of animals at different developmental stages (L1-L3, L4, and adults) was counted at 24, 48 and 72 h. The results were expressed as the number of animals at a given stage x 100/ Total number of living worms at that time point. This experiment was independently repeated three times.

#### Larvicidal activity assays

To study the effect of DII on larvae, isolated eggs (see method above) were incubated in M9 buffer without food overnight. This protocol leads to a homogenous population of L1 worms that were subsequently seeded on 35 mm plates containing DII and DMSO as a control. Three concentrations of drugs were used (100, 300 and 600 μM) and each concentration was repeated in triplicate. After 24, 48 and 72 h alive worms were scored and the results were expressed as the percentage of living worms related to the initial number of L1 animals. This experiment was independently repeated three times.

### Statistical analysis

One-way analysis of variance (ANOVA) was used to test statistical differences among treatment groups (for more than two groups) or *t*-test (for two groups). If the group means were statistically different (*p*< 0.05), we used the Holm-Sidak method multiple comparison tests to determine significant differences between groups. Dose-response curves were fitted using Prism 6 (GraphPad Software Inc., La Jolla, CA, USA) to a Hill equation with four (Eq 1) or five (Eq 2) parameters depending on the symmetry of each curve. No parameters were constrained. Hill-slope, EC50, LC50 and R^2^ values are provided in each figure legend.

$$\begin{array}{ll} 4\text{PL} & \text{Y}=\text{min}+\left(\text{max}-\text{min}\right)/[1+({\left(\text{X}/\text{EC}50\right)}^{-\text{Hill}-\text{slope}})] \end{array}$$ (1)

$${\begin{array}{ll} 5\text{PL} & \text{Y}=\text{min}+\left[\left(\text{max}-\text{min}\right)/\left(1+{\left(\text{X}/\text{Xb}\right)}^{-\text{Hill}-\text{slope}}\right)\right] \end{array}}^{\text{S}}$$ (2)

Xb=EC50*10[(1/Hill-slope)*log((2^(1/S))−1)]

## Results

### Anthelmintic activity of imidazole derivatives

**Fig 1:**
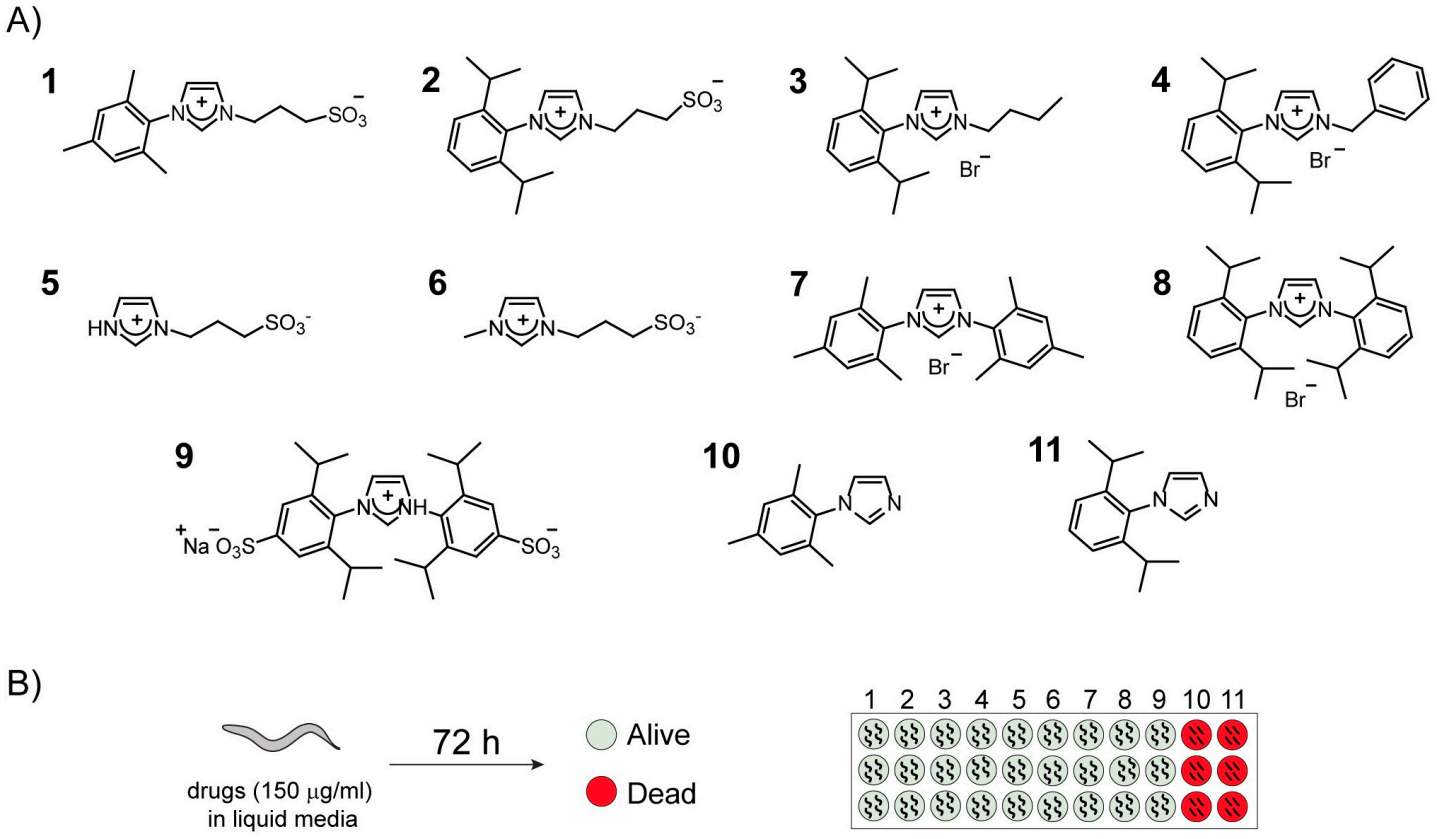
Anthelmintic activity screening of new imidazole-derivative compounds. (A) Structures of imidazolium salts and neutral compounds synthesized (compounds **1–11**). (B) To evaluate nematicidal effect L4/young adult *C*. *elegans* were exposed to the compounds (150 μg/ml) for 72 h. Animal survival was subsequently evaluated. Only compounds **10** and **11** induced animal death.

To contribute to the development of novel anthelmintic drugs, we here evaluated the nematicidal activity of 11 imidazole-derivatives on *C*. *elegans* (Fig 1A). The structural design of these compounds was directed to evaluate two properties: water solubility, which may be critical concerning absorption through biological barriers, and steric hindrance which may play a relevant role in the interaction with putative targets. Nine imidazolium salts (**1–9**) and two neutral imidazoles (**10** and **11**) were synthesized from anilines. Imidazole derivatives differing in water solubility and steric hindrance were synthesized by introducing polar groups and bulky residues composed of an aryl or benzyl group substituted by methyl or isopropyl groups (S1 Fig). Compounds **1**, **2**, **5**, **6** and **9** contain one or two sulfonated groups conferring solubility in water. The remaining compounds have a bromide or chloride as counter anions (Fig 1A). The overall yields of the isolated product were between 20% and 98%. All synthesized compounds were solid, air and moisture stable, and could be stored for long periods of time (see Material and methods).

We exposed 100–150 young adult worms to these compounds in liquid medium (S-Basal) containing the bacteria *E*. *coli* OP50 as a food source. After 72 h at 20°C, we analyzed worm survival (Fig 1B). Animals were considered dead if they did not move upon gentle prodding in the anterior part of the body. The drug concentration chosen for this screening was 150 μg/ml, which is similar to those used in other screening studies [40]. The corresponding molar concentration for each compound is detailed in Materials and Methods. Depending on their solubility properties, the compounds were dissolved either in water or DMSO (up to 0.75% final concentration).

Under these conditions, we found two compounds, **10** and **11**, that produced 100% worm death (Fig 1B). Compounds **10** and **11** are mesityl-imidazole (MI) and diisopropylphenyl-imidazole (DII), respectively. These compounds are intermediates in the synthesis of imidazolium salts **1–4**. Unlike the rest of the drugs tested, MI and DII are neutral imidazoles with one substituted nitrogen, while the other nitrogen conserves basic properties (Fig 1A).

**Fig 2:**
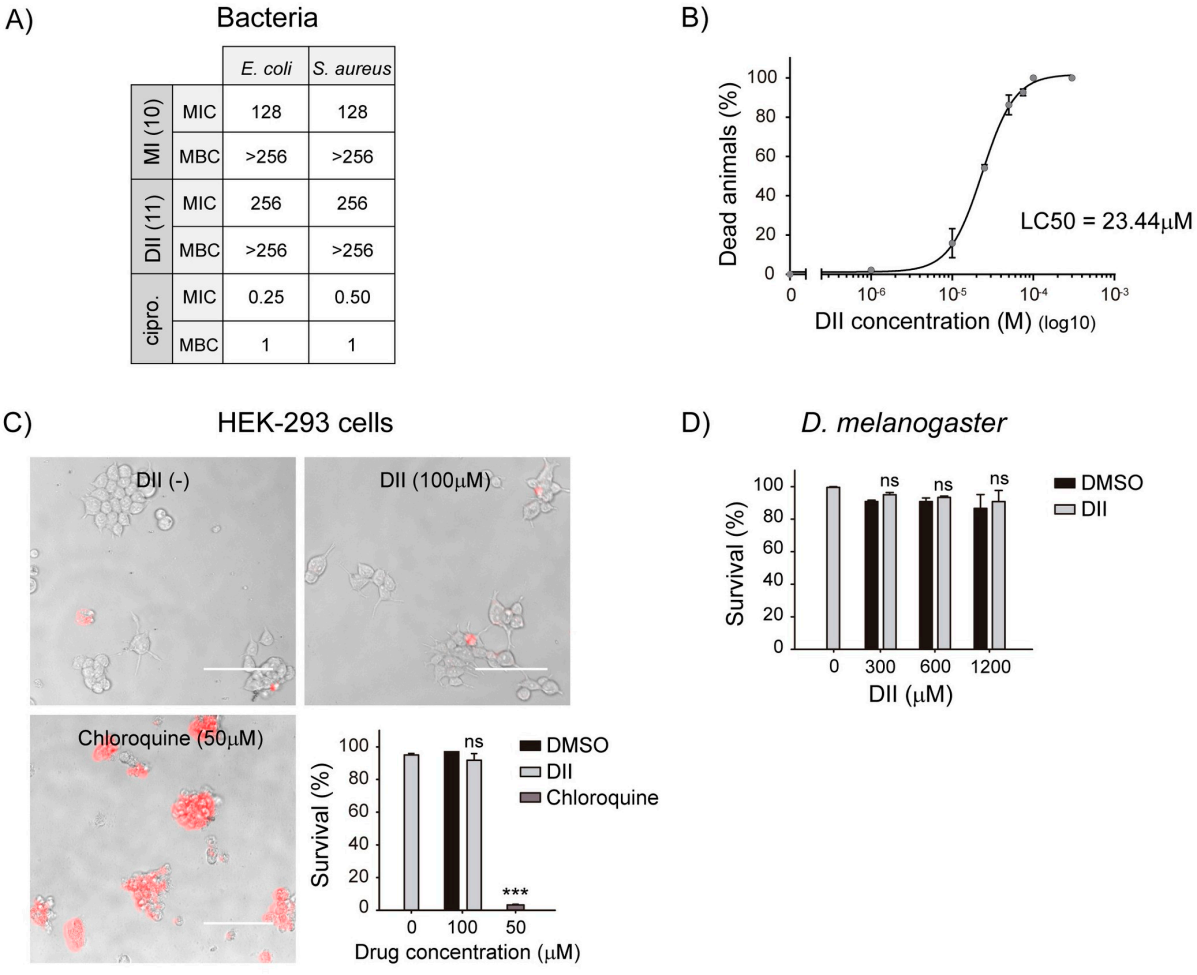
Selective toxicity of DII. (A) The effect of MI (**10**) and DII (**11**) in bacteria was evaluated using *E*. *coli* and *S*. *aureus* cultures. Minimum inhibitory concentration (MIC) and minimum bactericidal concentration (MBC) values were obtained using microdilution methods. As control of antibiotic activity ciprofloxacin (cipro.) was used. (B) DII lethal dose curve. 40 L4 wild-type worms were exposed to a range of DII concentrations and animal death was evaluated after 96 h of exposure. Data was fitted with a 4PL curve. LC50 = 23.44 ± 1.06 μM, Hill slope = 2.15 ± 0.25, R^2^ = 0.99. Each concentration point represents the mean value ± SEM of three independent experiments. (C) DII effect on human cell cultures was evaluated using HEK-293 cells. Cells were exposed to DII (0–100 μM) and after 96 h of incubation, cell death was quantified using Propidium Iodide (PI) staining. Red staining accounts for dead cells. DMSO (0.1%) and chloroquine (50 μM) were used as negative and positive control, respectively. Bar scale: 100 μm. Results are presented as mean ± SEM (ns: no statistically significant, p > 0.05, ***p<0.001; *n* = 3) (D) DII effect on *Drosophila melanogaster*. Fly larvae were exposed to DII (300–1200 μM) until they left the food to pupariate. Larval survival was calculated as the percentage of larvae that reached the pupal stage. Results are presented as mean ± SEM (ns: no statistically significant, p > 0.05; *n* = 3).

During the screening assays, we qualitatively noticed that OP50 growth was inhibited in wells containing MI. This effect was not observed with DII. To quantify this observation, we determined minimum inhibitory concentration (MIC) and minimum bactericidal concentration (MBC) of both compounds in *E*. *coli* cultures (Fig 2A). The MIC values obtained were 128 μg/ml (0.68 mM) and 256 μg/ml (1.11 mM) for MI and DII, respectively. Similar values were obtained when we performed these assays on *S*. *aureus*, a Gram-positive bacteria (Fig 2A). These results indicate that at the concentration tested in the screening, MI does not only affect worm survival but also inhibits bacterial growth (Figs 1 and 2A). As we are here screening for specific anthelmintic activity, we restricted our further analysis to compounds that appear to exert nematicidal action without obvious lethal effects on other organisms. Therefore, we focused our studies only on DII, relegating the evaluation of MI as a potential anthelmintic drug.

To start the analysis of DII as a potential valuable anthelmintic compound, we examined whether DII is able to induce *C*. *elegans* death at lower concentrations than that used in the screening (~ 600 μM). To this end, we exposed L4 animals to different DII concentrations for 96 h (Fig 2B). We found a lethal effect that was clearly dependent on the concentration of DII used. The LC50 value was 23.44 ± 1.06 μM (Fig 2B) which is similar to those reported for established anthelmintic drugs such as albendazole [41, 42].

To further explore the specificity of DII lethal effect on nematodes, we assessed its toxicity in a human cell culture (HEK-293) and *Drosophila melanogaster* larvae. Similar to our experiments in *C*. *elegans*, we exposed HEK-293 cells to DII (100 μM) for 96 h. Cell viability was subsequently quantified using the fluorescent dye propidium iodide (PI). Unlike its lethal effects on *C*. *elegans*, we found that DII concentrations up to 100 μM, do not affect cell viability (Fig 2C).

Furthermore, we evaluated the effect of DII on the viability of larvae of the fly *Drosophila melanogaster*. To this end, we fed age-synchronized larvae with food containing DII at different concentrations (300–1200 μM) and scored the proportion of animals that reached the pupa stage after approximately 40 h of exposure (Fig 2D, S2B Fig). Contrary to the lethal effects observed on *C*. *elegans*, we found that DII treatment does not affect *Drosophila* larval viability at the concentration range tested.

Taken together, our experiments demonstrate that DII exposure results lethal to *C*. *elegans*. In contrast, similar or even higher concentrations of this compound do not affect the viability of the prokaryotic organisms, mammalian cultured cells and insects tested. This specificity prompted us to evaluate it as a potential new anthelmintic.

### Molecular mechanism underlying DII anthelmintic activity

**Fig 3:**
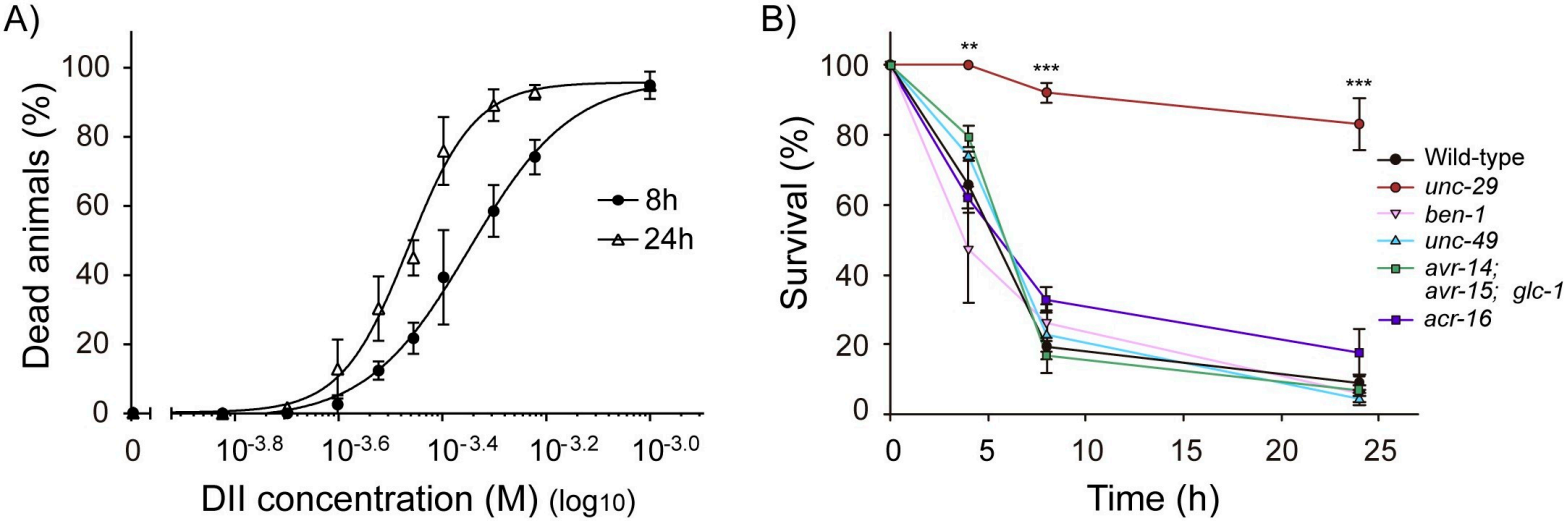
Nematicidal effect of DII on wild-type and mutant *C*. *elegans* strains. (A) DII lethal dose curves at 8 (black circle) and 24 (white triangle) h of exposure. ~40 L4 wild-type worms were exposed to a range of DII concentrations and animal death was evaluated at different time points. Data was fitted with a 4PL curve. (black circle) LC50 = 448.5 ± 1.1 μM, Hill-slope = 4.4 ± 0.9, R^2^ = 0.91 and (white triangle) LC50 = 342.3 ± 1.0 μM, Hill-slope = 6.7 ± 1.3, R^2^ = 0.95. Each concentration point represents the mean value ± SEM of three independent experiments (B) DII nematicidal effect on *C*. *elegans* mutant strains previously reported as resistant to currently used anthelmintic agents. ~80 L4 mutant animals were exposed to DII (600 μM) and worm survival was scored at each indicated time (4, 8 and 24 h). Only CB1072 *unc-29(e1072)I* strain was resistant to DII anthelmintic activity.‬ Results are presented as mean ± SEM. Statistical significance compared to wild-type worms (**p<0.01, ***p<0.001; *n* = 3).

To further characterize the lethal effect of DII on *C*. *elegans*, we tested whether this drug is capable of inducing worm death in a shorter timescale than 96 h. To this end, we analyzed the survival of L4 animals exposed to different concentrations of the drug (up to 1 mM) for 8 and 24 h (Fig 3A). LC50s were 448.5 ± 1.1 and 342.3 ± 1.0 μM for 8 and 24 h of exposure, respectively. As was observed for 96 h exposure assays, the DII concentrations that result lethal to *C*. *elegans*, do not affect viability on HEK-293 cells (S2A Fig). We also examined the recovery capacity of worms treated with DII (600 μM). To this end, those nematodes that survived to 4 and 8 h DII exposure were subsequently transferred to regular NGM plates. The fact that most of the DII-treated nematodes resumed normal growth suggests that DII effect is reversible (S3 Fig).

To gain insights into the molecular targets underlying DII nematicidal effect, we analyzed its lethality in several *C*. *elegans* mutant strains previously reported as resistant to currently used anthelmintics. The strains used, as well as their resistance profile, are detailed in Table 1.

**Table 1:** Strains used and their drug resistance profile.

Strain Genotype	Drug Phenotype
*ben-1(e1880)III*	Benzimidazoles resistance
*avr-14(ad1305)I; avr-15(vu227)V; glc-1(pk54)V*	Ivermectin resistance
*unc-49(e407)III*	Levamisole hypersensitivity
*unc-29(e1702)I*	Levamisole resistance
*unc-63(x26)I*	Levamisole resistance
*unc-38(e264)I*	Levamisole resistance
*lev-1(ok3201)IV*	Levamisole resistance
*lev-8(ok1519)X*	Levamisole resistance
*acr-16(ok789)V*	No Nicotine-sensitive AChR in body wall muscles

**Fig 4:**
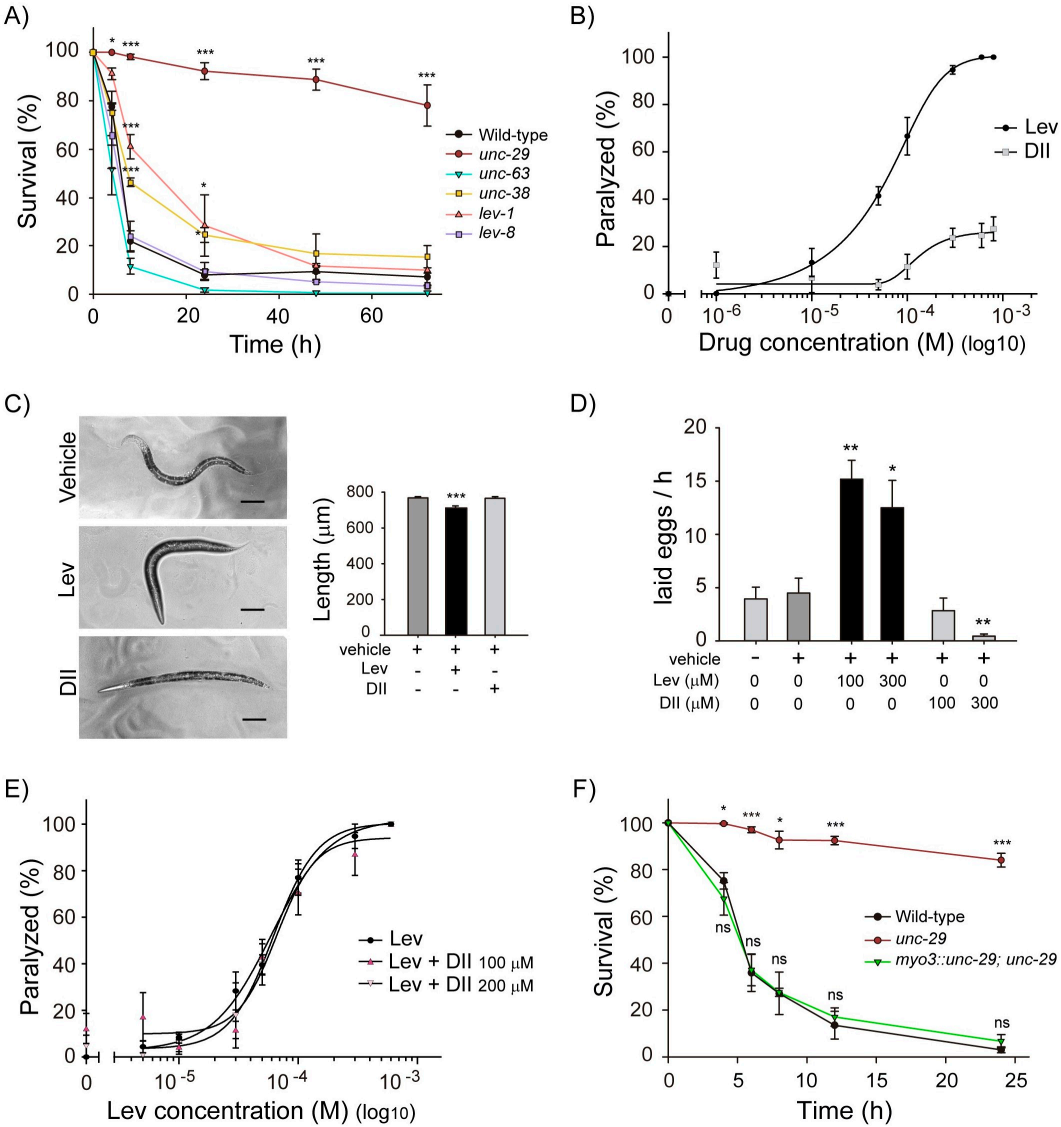
DII effect on L-AChR-deficient mutants. (A) DII-anthelmintic activity in different L-AChR subunit mutants. ~80 L4 mutant animals were exposed to DII (600 μM) and after the indicated time (4, 8, 24, 48 and 72 h) survival was scored. Results are presented as mean ± SEM. Statistical significance compared to wild-type worms (*p<0.05, **p<0.01, ***p<0.001; *n* = 3). (B) Acute paralysis assays. 30–40 L4 worms were exposed to different levamisole or DII concentrations (0–800 μM) and 10 minutes later paralysis was evaluated. Data was fitted with a 5PL curve. (black circle) EC50 = 63.6 ± 1.1 μM, Hill-slope = 2.39 ± 1.37, R^2^ = 0.97 and (grey square) Hill-slope = 1.96 ± 2.31, R^2^ = 0.49. Each concentration point represents the mean value ± SEM of three independent experiments. (C) Body length measurement after levamisole and DII (600 μM) 2 h treatment. Results are presented as mean ± SEM (***p<0.001; n = 20) Bar scale: 100 μm. (D) Egg laying rate in levamisole and DII-treated animals. 12 gravid worms were incubated for 1 h with the drugs (100 and 300 μM) and the number of laid eggs was counted. Results are expressed as the mean number of laid eggs per animal in 1 h ± SEM (*p<0.05, **p<0.01; *n* = 4). (E) Levamisole dose-response curves in presence of DII. Animals were exposed to a range of levamisole concentrations (1–600 μM) in the presence of constant DII concentrations (100 or 200 μM). After 10 minutes of incubation, paralysis was evaluated. No differences were observed among the three dose-response curves. Data was fitted with a 4PL curve. (black circle) EC50 = 60.00 ± 1.12 μM, Hill-slope = 1.78 ± 0.36, R^2^ = 0.91, (dark pink triangle up) EC50 = 66.03 ± 1.15 μM, Hill-slope = 2.65 ± 0.81, R^2^ = 0.90, (light pink triangle down) EC50 = 62.25 ± 1.14 μM, Hill-slope = 2.36 ± 0.61, R^2^ = 0.93. Each concentration point represents the mean value ± SEM of three independent experiments. (F) UNC-29 muscle expression restores DII sensitivity. *unc-29(e1072)I* animals were injected with P*myo-3*::*UNC-29;* to restore muscle UNC-29 expression. Wild-type, *unc-29* null mutants and muscle rescue were exposed to DII (600 μM) and worm survival was evaluated at different time points (4, 6, 8, 12 and 24 h). Data are presented as mean ± SEM. Statistical significance compared to wild-type worms (*p<0.05, ***p<0.001, ns: no statistically significant, p > 0.05; *n* = 3).

We found that CB1072 *unc-29(e1072)I* is extremely resistant to the nematicidal effect of DII (Fig 3B, S4 Fig). CB1072 contains a null mutation in *unc-29*, a non-alpha nematode AChR subunit, and it has been largely described as a levamisole-resistant strain [25, 26, 43]. In muscles, UNC-29 coassembles with UNC-38, UNC-63, LEV-1, and LEV-8 to form the levamisole-sensitive AChR (L-AChRs) [38, 43–46]. Mutations in *unc-29*, *unc-38* and *unc-63* preclude the functional expression of this receptor, therefore inducing strong levamisole resistance [25, 43]. On the other hand, mutations in *lev-1* and *lev-8* do not completely abolish L-AChR activity leading to mild resistance to these classic cholinergic anthelmintics [25, 47, 48]. Unexpectedly, mutant strains in *unc-63 and lev-8* are as sensitive to DII as the wild-type (Fig 4A and S4 Fig). Moreover, although mutants in *unc-38* and *lev-1* are slightly resistant to DII at short exposure times, they do not exhibit significant differences with the wild-type after 48 h of exposure (Fig 4A and S4 Fig). This strongly suggests that DII mechanism of action differs from that reported for classical cholinergic anthelmintics such as levamisole.

To confirm the hypothesis that DII and levamisole act differently, we compared the effect of these two drugs on *C*. *elegans* locomotion, body length and egg laying rate. Levamisole has been largely described as a very potent agonist of muscle AChRs in roundworms (Reviewed in [18]). After a brief exposure, levamisole causes sustained depolarization of nematode muscle membrane leading to hypercontraction and paralysis [17, 49, 50]. In addition, levamisole-activated AChRs in vulval muscles stimulate egg laying [51]. We evaluated acute paralysis after 10 min of exposure to a wide range of drug concentrations (1–800 μM). As previously reported, we observed that levamisole induced a concentration-dependent spastic paralysis (EC50 = 63.64 μM, Fig 4B). At 600 μM 100% levamisole-treated animals were completely paralyzed. Remarkably, DII did not induce significant animal paralysis. Even at the highest concentration tested (800 μM), only 25% of DII-treated animals were immobilized (Fig 4B). Unfortunately, we could not test higher DII concentrations due to DMSO effects on locomotion. Since the cuticle is an important physical barrier that impairs drug absorption, we also analyzed DII effects using a cut worm model where the effects of nicotinic ligands are achieved at lower concentrations in comparison to intact animals [26, 39]. As previously reported, we found that in cut specimens paralysis is achieved at very low levamisole concentrations (S5A Fig). Nevertheless, high concentrations of DII (600 μM) fail to produce significant paralysis even in cut specimens (S5A Fig). In addition, we analyzed drug-induced paralysis in the strain CB407, which is a null mutant of the muscle GABA receptor, UNC-49 (Table 1). This mutant background leads to hypersensitivity to the paralyzing effect of L-AChR agonists such as levamisole or pyrantel [52]. We found that even in this hypersensitive background no paralyzing effects were induced by DII (S5B Fig).

Furthermore, we also measured body length after drug exposure (600 μM). As previously reported for nAChR agonists [53], we found that levamisole exposure leads to worm shrinkage as a consequence of massive body wall muscle contraction (Fig 4C). On the contrary, no significant changes were observed in body length after DII treatment (Fig 4C).

To compare the effects of levamisole and DII on egg laying rate, we exposed gravid animals to 100 and 300 μM of either levamisole or DII for 1 h and quantified the number of laid eggs (Fig 4D). Since neither levamisole nor DII affected worm viability at these concentrations in 1 h, we can rule out that, in our experimental conditions, animal lethality could affect egg laying. As expected, levamisole treatment significantly increased the number of laid eggs in 1 h (3.8-fold and 3.2-fold more laid eggs than controls for 100 and 300 μM levamisole, respectively) (Fig 4D). In contrast, DII treatment did not produce an increase in the number of laid eggs at 100 μM (Fig 4D). Moreover, a 3.6-fold reduction in the number of laid eggs was observed at 300 μM. These results further demonstrate that DII is not a traditional agonist of L-AChRs as levamisole or pyrantel.

We also evaluated the possibility that DII antagonizes levamisole action. To this end, we exposed animals to increasing concentrations of levamisole (from 1 to 600 μM) and constant concentrations of DII (100 and 200 μM). Animal paralysis was scored after 10 minutes of drug exposure. The presence of DII led to no significant changes in the dose-effect curve of levamisole in intact animals (Fig 4E). Moreover, DII did not affect the response to levamisole in cut specimens either (S5C Fig). To evaluate whether longer exposures to DII could affect levamisole response, we repeated these dose-effect curves in animals that were pre-incubated with 100 μM DII for 24 h. We found no differences in the sensitivity to levamisole between pre- and untreated animals (S5D Fig). Taken together, these results strongly suggest that DII does not antagonize L-AChRs.

Previous reports demonstrated that *unc-29* is mainly expressed in muscles [43, 54, 55]. To confirm that the nematicidal action of DII depends on the muscle expression of *unc-29* we analyzed DII-induced lethality in the OAR61 (*myo-3*::*unc-29; unc-29*(*e1072)I*) transgenic strain, that expresses *unc-29* exclusively in body wall and vulval muscle cells. We found that muscle expression of *unc-29* completely restores DII-mediated death to wild-type levels (Fig 4F and S4 Fig). Therefore, we can conclude that DII targets a novel UNC-29-containing AChR expressed in *C*. *elegans* muscle which is different from the classic muscle L-AChR. Further experiments are needed to elucidate the stoichiometry and function of this receptor and the mechanism underlying DII action.

Interestingly, we also found that DII exposure decreases pharyngeal pumping rate of wild-type animals. (S6 Fig). In agreement with previous reports showing that *unc-29* is not involved in the control of pharyngeal pumping [56], DII-dependent pharyngeal inhibition was also observed in *unc-29* null mutants. Although this demonstrates that DII can act through different targets, the fact that *unc-29* mutants can survive DII exposure suggests that the pumping rate reduction does not mediate DII lethal effect.

### DII affects *C*. *elegans* immature stages

One of the main goals of anthelmintic therapies is to eliminate the parasite independently of its developmental stage. Nevertheless, immature stages are generally more resistant to classic nematicidal drugs as levamisole [18].

**Fig 5:**
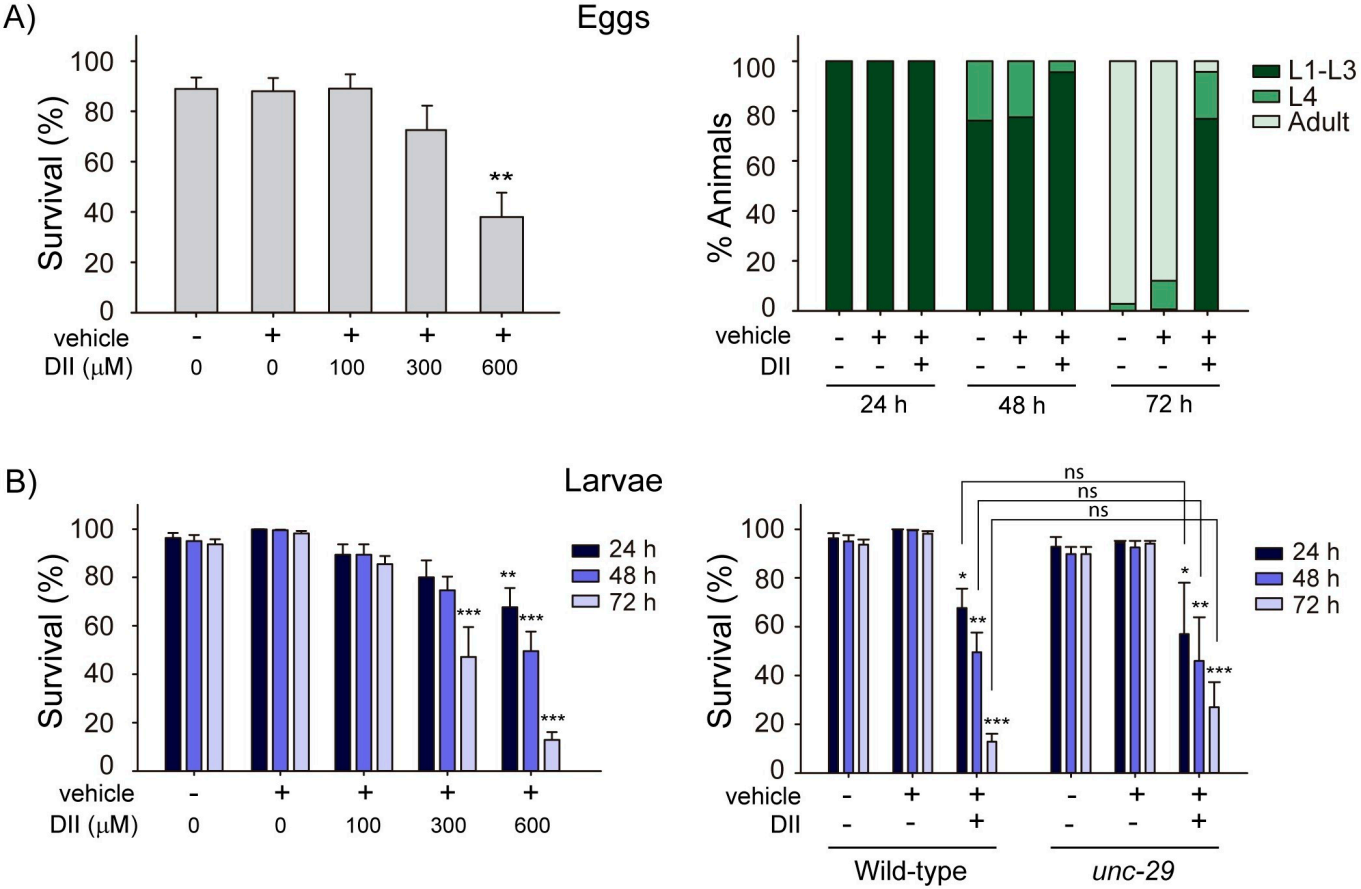
DII anthelmintic activity in *C*. *elegans* immature stages. (A) Left. Isolated eggs were exposed to DII (0–600 μM). After 48 h of exposure, living animals were counted. Survival percentages are relative to the number of eggs at the beginning of the assay. Data are presented as mean ± SEM. Statistical significance compared to control (**p<0.01; *n* = 3) Right. DII effect on *C*. *elegans* developmental rate. Eggs were exposed to DII (600 μM) and animal stages were evaluated at each indicated time point (24, 48 and 72 h). L1-L3: early larval stages, L4: last larval stage. Animal stage percentages are relative to the number of living animals at the indicated time point. (B) Left. L1 larvae were exposed to DII (100, 300 and 600 μM) and after 24, 48 and 72 h animal viability was evaluated. Right. DII (600 μM) larvicidal effect on *unc-29* mutant larvae. Data are presented as mean ± SEM. Statistical significance compared to control at the same time point (*p<0.05, **p<0.01, ***p<0.001, ns: no statistically significant, p > 0.05; *n* = 3).

To directly evaluate DII effects on *C*. *elegans* early developmental stages, we exposed eggs to different concentrations of DII (100, 300 and 600 μM). We found that after 48 h of exposure the number of viable animals (relative to the initial number of eggs seeded) was significantly reduced (Fig 5A, left). Moreover, while most non-treated eggs lead to adult animals after 72 h, less than 5% of survivors were adults on DII-containing plates (Fig 5A, right). Therefore, we can conclude that DII treatment affects immature stages and delays nematode development.

As in the previous experiment, animals were exposed to DII during the whole assay, we could not discriminate if the reduction in viable animals was due to DII effect on eggs, on larval stages or both. To unequivocally evaluate the potential ovicidal effect of DII we isolated eggs and scored hatching by video recording in the absence and presence of the drug. Similar to control conditions, 100% of DII-exposed eggs hatched within 12 hours (S1 and S2 movies). This suggests that the effect observed in immature stages is due to a larvicidal effect of DII.

To further confirm this larvicidal effect, we isolated wild-type animals at the L1 larval stage (see Methods) and scored survivors after 24, 48 and 72 h of DII exposure. We found a significant decrease in larval viability in DII-treated animals (Fig 5B, right). These results clearly demonstrate that apart from being lethal to adult nematodes, DII also affects larval stages.

Does the larvicidal effect of DII also depend on UNC-29? To address this, we analyzed the effect of DII (600 μM) on larval stages of *unc-29* null mutants. Unexpectedly, we found that *unc-29* null mutants are as sensitive to the larvicidal effects of DII as the wild-type strain (Fig 5B, left). Moreover, DII-dependent developmental delay was also found in *unc-29* null mutant animals (S7 Fig). These results show that, unlike our findings in adult worms, the larvicidal effect of DII does not involve UNC-29. Similar to *unc-29* null mutants, *unc-38*, *unc-63*, *lev-8*, and *lev-1* larvae are also sensitive to DII (S8 Fig). Further experiments are needed to asses if the larvicidal effect depends on unidentified AChRs or if it involves other types of targets.

Taking together, we here found a novel drug with anthelmintic effects in the nematode model *C*. *elegans*. The targets underlying the nematicidal effect of DII vary depending on the developmental stage: while it entirely depends on the muscle AChR subunit UNC-29 in adults, its larvicidal effect involves a different target that needs further examination to be identified.

## Discussion

In this work, we screened imidazole-derivatives on *C*. *elegans* to identify novel compounds with potential anthelmintic effects. We found a compound, diisopropylphenyl-imidazole (DII), that exerts nematicidal effects on both adult and immature stages of *C*. *elegans*. Interestingly, while DII lethal effects on adults depend on the non-alpha nicotinic receptor subunit UNC-29, the receptor target is not the classical levamisole-sensitive AChR. Moreover, we also found that the larvicidal effect of DII does not depend, at least exclusively, on UNC-29.

Among all the compounds tested, only mesityl imidazole (MI, compound **10**) and diisopropylphenyl-imidazole (DII, compound **11**) were active against *C*. *elegans*. Both are neutral molecules with no charge on the imidazole ring and both have one nitrogen atom free of substituents which can interact with the environment as a weak base. Both compounds are planar molecules with one substituent in one nitrogen atom, the substituent of DII bulkier with two isopropyl groups being able to rotate free. These chemical features could explain the differences in activity between MI and DII. As a direct result of the screening, it was clear that the neutral imidazole ring improved nematicidal effect against *C*. *elegans*. This is not surprising due to the fact that lipophilic drugs pass better through the nematode cuticle.

Specificity is a crucial requirement for drugs intended to treat pathogenic infections as it reduces the chances of adverse reactions on the host. We found that DII does not impair the viability of *D*. *melanogaster* and mammalian-derived culture cells, at similar or even higher concentrations than those required to kill *C*. *elegans*. This demonstrates that DII does not affect vital biomolecules conserved throughout the animal kingdom.

Drugs inducing death in *C*. *elegans* are extremely likely to also kill parasitic nematodes [30, 57], thus supporting the potential of DII as a promising anthelmintic agent to treat parasitosis. Given the impermeability of *C*. *elegans* cuticle, higher drug concentrations are generally needed to kill this free-living nematode in comparison with parasitic roundworms [41]. For instance, micromolar concentrations of albendazole, pyrantel, nitazoxanide, and ivermectin are enough to kill parasitic nematodes in 24 h [41], whereas less than 25% of *C*. *elegans* death is achieved even at millimolar concentrations [39, 41]. In contrast, we found that 24 h of exposure to micromolar DII concentrations induces 100% lethality to the “impermeable” nematode *C*. *elegans*. It should be noted that the anthelmintic efficiency of levamisole, pyrantel or ivermectin against gastrointestinal parasites does not depend on its lethality but on their paralyzing capacity at low micromolar concentrations. Nevertheless, when compared with drugs that exert their therapeutical effect by inducing nematode death (e.g albendazole), DII appears to be a promising efficient nematicidal agent.

DII exerts its nematicidal effects on both mature and immature stages of *C*. *elegans*. Regarding its effect on adult nematodes, we found that lethality depends on the muscular expression of UNC-29, a non-alpha nicotinic receptor subunit. Similar to UNC-38 and UNC-63, UNC-29 has been largely reported to be an essential constituent of the muscle levamisole-sensitive AChR (L-AChR), a classic target for anthelmintic compounds such as levamisole and pyrantel [38, 43–45]. These drugs potently activate L-AChRs leading to spastic muscle paralysis [18]. Mutations in *unc-29*, *unc-38* or *unc-63* lead to strong levamisole resistance [25]. In contrast, *lev-1* and *lev-8* (non-essential subunits of L-AChRs) mutant animals, are only partially resistant to levamisole [25]. We here compile strong evidence that allow us to conclude that DII acts through a mechanism different from that of classical cholinergic anthelmintics: i) Although they are strongly resistant to levamisole [25, 44], strains mutated in *unc-63* are as sensitive to DII as wild-type animals. Moreover, *lev-8* mutants also show high DII sensitivity, ii) the strongly levamisole-resistant strain CB904 (*unc-38(e264)I)* is completely sensitive to DII exposures longer than 24 h and it is only slightly resistant to shorter exposure times, iii) unlike levamisole and other nAChRs agonists such as morantel or pyrantel, DII does not cause acute paralysis, body shrinkage or increased egg laying rate, iv) the presence of DII does not produce significant shifts to levamisole doses/effect curves and v) *unc-49* null mutant background, which is associated with levamisole hypersensitivity, does not lead to significant alterations in DII sensitivity. These observations raise the tantalizing possibility that UNC-29 could be a constituent of an unidentified muscle cholinergic receptor in *C*. *elegans*, different from the traditional levamisole-sensitive AChR.

In *C*. *elegans*, whole-cell voltage clamp recordings from body wall muscle revealed that perfused ACh elicits detectable activity from two types of AChRs: namely L-AChR, formed by UNC-29, UNC-38, UNC-63, LEV-1, and LEV-8, and a homopentameric nicotine-sensitive AChR (N-AChR), formed by the alpha7-like subunit ACR-16 [43, 58, 59]. No response to ACh was detected on adult body wall muscle of *unc-63; acr-16* double mutants [59]. Nevertheless, reports using tandem affinity purification and electrophysiological approaches suggest that other functional AChRs may exist in *C*. *elegans* muscles [60, 61]. Moreover, while the synaptic expression of LEV-8 and UNC-38 clusters in the anterior ends of ventral and dorsal nerve cords, there is no detectable LEV-8 expression in the rest of the body, thus suggesting that AChR composition could differ among muscle cells [62]. Since *in vivo* recordings are usually performed on muscles from the ventral-medial region [63], it is possible that muscle AChRs expressed in other regions could not be detected. Further supporting evidence for the existence of unidentified AChRs relies on the muscle expression of several nAChRs subunits that do not form the native N- or L-AChRs (reviewed in [64]). It could be possible that UNC-29 co-assembles with some of these subunits to form a functional muscle AChR, different from “traditional” L-AChRs.

Since *unc-38* and *lev-1* mutants are slightly resistant to DII it could be that these subunits are also part of this novel receptor. In a mutant background, other subunits could replace them with a minimal impairment of DII sensitivity. Subunit replacement has been described as a common mechanism for different AChRs in knockout animals [65]. Alternatively, these two subunits could form a minor population of DII-sensitive AChR (different from that containing UNC-29). It will be extremely interesting to confirm the existence of this/these unidentified receptor/s and to determine its/their stoichiometry, function, and biophysical and pharmacological properties.

Based on their response to anthelmintic drugs, multiple muscle AChRs subtypes have been described in parasitic nematode species [64, 66, 67]. Consistent with our hypothesis regarding *C*. *elegans*, UNC-29 appears to be part of multiple muscle AChRs in *Brugia malayi*, *Oesophagostomum dentatum*, and *Ascaris suum* [64, 66]. Our results suggest that the great variety of AChRs subtypes expressed in muscle could be a shared feature between parasitic nematodes and *C*. *elegans*. Unlike mammals, where mutations in muscle AChR are usually linked with severe movement defects [68], several nematode muscle receptors are not crucial for animal motility. The functional role of such a diversity in nematode muscle AChRs is still intriguing.

Our experiments do not allow to elucidate if DII acts through activation or inhibition of this UNC-29-containing receptor. Since UNC-29 null mutants are viable, the hypothesis that DII activates this receptor, rather than inhibiting it, could be considered more likely. Nevertheless, paraherquamide is thought to exert its anthelmintic effect by antagonizing L-AChRs, whereas worms with mutations in L-AChR subunits are viable [1]. It is possible that genetic mutations, but not pharmacological inhibition of AChRs trigger compensatory mechanisms to attenuate detrimental consequences, therefore allowing animal viability. Unexpectedly for a drug that acts through muscle AChRs, DII does not significantly affect nematode motility. This is similar to the effect of derquantel, an antagonist of the morantel-sensitive AChR, on *Brugia malayi* [66]. Since most of the cholinergic agonists (independently of the AChR subtype they activate in muscle) leads to paralysis [1], it could be speculated that, similar to derquantel, DII could act as an antagonist of a muscle AChR yet to be identified. Alternatively, DII could exert its effect through positive or negative allosteric regulation or by modulating desensitization of an UNC-29-containing receptor. Further experiments are needed to unequivocally elucidate DII mechanism of action.

We demonstrated that DII is effective against larval stages of *C*. *elegans*. DII induces worm death and delays development arresting animals in initial larvae stages. These properties are a clear advantage given that young animals do not reach fertility and will die before the chance of leaving progeny.

Strikingly, *unc-29* null mutant larvae are as sensitive to DII as the wild-type strain, strongly suggesting that a different DII target is expressed only at early developmental stages. UNC-29 is expressed in *C*. *elegans* muscle from L1 to adult stage [43]. From our experiments, we cannot rule out that DII larvicidal effects arise from its action on two different types of targets: UNC-29-containing AChRs and an unknown larval specific target. The lethality observed in *unc-29* null mutant larvae could arise for DII action on this second larval-specific target. Nevertheless, it has been largely reported that the efficacy of anthelmintics that target AChRs is reduced in larval stages [25, 69] probably due to changes in nematodes subunit expression pattern during development as occurs in mammals [70, 71]. Moreover, at similar DII concentrations the timescale for lethality in larvae is longer than in adults, thus suggesting that the larvicidal effect depends mainly on a different mechanism of action. In the future, it will be extremely interesting to identify this larval specific DII target.

The nematicidal effect of DII on both immature and mature nematode stages could offer an improvement to classical antiparasitic drugs such as levamisole, pyrantel or morantel which are less effective against immature stages [69]. Nowadays, combinations of different anthelmintic drugs that act through different targets, such as levamisole and mebendazole, are frequently administered. The double-aim of this association is to maintain nematode control even in the presence of resistant worms and to delay resistance development [72, 73]. The fact that DII produces lethality through two (or more) different targets may impair resistance development. These features could provide a significant advantage even over relatively new anthelmintic drugs, such as tribendimidine and monepantel, whose nematicidal effects appear to depend on a single target [27, 29].

In conclusion, we demonstrated that DII fulfills the major criteria necessary for the development of a novel anthelmintic: phylogenetic specificity and a novel biochemical mode of action. These properties together with the fact that most molecules that kill *C*. *elegans* have been proved to be lethal to parasitic nematodes [30], shape this imidazole-derivative compound as a strong anthelmintic candidate that deserves further investigations using parasite nematodes *in vitro* and in their natural hosts.

## Supporting information

S1 Fig Synthetic route and water (w) or DMSO solubility. (TIF)

S2 Fig Selective toxicity of DII. (A) DII effect on human cell cultures was evaluated using HEK-293 cells. Cells were exposed to DII (0–600 μM) and after 8 h of incubation, cell death was quantified using Propidium Iodide (PI) staining. Untreated and chloroquine-treated (200 μM) cells were used as a negative and positive control, respectively. Upper: PI staining microphotographs. Red staining accounts for dead cells. Bar scale: 100 μm. Bottom: Cell survival quantification. Results are presented as mean ± SEM (ns: no statistically significant, p > 0.05, ***p<0.001; n = 3) (B) Positive control for *Drosophila* larval viability assay. Fly larvae were exposed to Sodium Azide (0.5–2 mM) until they left the food to pupariate. Larval survival was calculated as the percentage of larvae that reached the pupal stage. Results are presented as mean ± SEM (ns: no statistically significant, p > 0.05, *p<0.05, ***p<0.001; n = 3).
(TIF)

S3 Fig Recovery from DII exposure. ~100 L4 worms were transferred to NGM plates containing DII (600 μM). After 4 and 8 h of exposure, survivors were transferred to regular NGM plates. Worm viability was scored 24 h later. Untreated worms were considered as 100% of viability (discontinued line). Results are presented as mean ± SEM. Statistical significance compared to untreated worms (ns: no statistically significant, p > 0.05; n = 3).
(TIF)

S4 Fig Resistance of wild-type and mutant *C*. *elegans* strains to DII long exposure. ~40 L4 mutant animals were exposed to DII (50 μM) for 96 h. Worm survival was subsequently scored. Only *unc-29* null mutant strain was resistant to DII anthelmintic activity.‬‬‬ Results are presented as mean ± SEM. Statistical significance compared to wild-type worms (ns: no statistically significant, ***p<0.001; n = 3).
(TIF)

S5 Fig Comparison between DII and levamisole effects. (A) Acute paralysis assays. Paralysis was scored in intact and cut adult worms (see Methods) exposed to levamisole (10 μM) or DII (600 μM) for 10 minutes. DII is unable to induce paralysis even in cut animals. Results are presented as mean ± SEM. Statistical significance compared to uncut worms (ns: no statistically significant, **p<0.01; n = 3). (B) Acute paralysis assays on CB407 *unc-49(e407)III* mutant strain. 30–40 young adult worms were exposed to each drug (100 μM levamisole or 100 μM DII). After 10 minutes, the number of paralyzed animals was scored. As expected CB407 strain is hypersensitive to levamisole. No significant differences between wild-type animals and CB407 strains were observed in DII-treated animals. Data are presented as mean ± SEM. Statistical significance compared to wild-type worms (ns: no statistically significant p>0.05 **p<0.01; n = 4). (C) Effect of DII on levamisole-induced paralysis in cut animals. Cut adult worms were exposed to levamisole (3, 5, 20 μM) alone and in the presence of two different concentrations of DII (5 and 15 μM). Similar to the observations in intact animals, the presence of DII does not impair levamisole action. Data are presented as mean ± SEM. Statistical significance compared to levamisole (Lev) treated worms. (ns: no statistically significant p>0.05; n = 3) (D) Levamisole dose-response curves after long pre-exposure to DII. Animals were exposed to DII (100 μM) for 24 h. After this treatment, they were exposed to a range of levamisole concentrations (1–600 μM) in the presence of DII (100 μM). Control curves were performed similarly, without the addition of DII neither in the preincubation nor in the paralysis assay. Data are fitted with a 4PL curve. (Black circle) EC50 = 82.43 ± 1.23 μM, Hill slope = 1.23 ± 0.26, R^2^ = 0.95. (Pink triangle) EC50 = 82.22 ± 1.39 μM, Hill slope = 1.38 ± 0.54, R^2^ = 0.88. Each concentration point represents the mean value ± SEM of three independent experiments. No differences were observed between pre-exposed and control animals.
(TIF)

S6 Fig DII effects on pharyngeal pumping. Wild-type and *unc-29* young adult worms (24 h past L4) were transferred to bacteria-seeded NGM plates containing different DII concentrations. After two hours of DII exposure, the number of contractions in the terminal bulb of the pharynx (pumps per minute) was counted using a stereomicroscope at 50x magnification. DII inhibits the pharyngeal pumping. However, the fact that this inhibition also occurs in *unc-29* mutants suggests that the effect on pumping rate does not underlie DII nematicidal action. Bars represent the mean ± SEM from n = 20 animals per condition. Statistical differences compared to the non-treated condition (0 μM DII) within the same strain (ns: no statistically significant p>0.05, ***p<0.001).
(TIF)

S7 Fig Developmental rate of DII treated *unc-29* mutant larvae. Isolated eggs were exposed to DII (600 μM) and at each time point (24, 48 and 72 h), animal developmental stages were scored. L1-L3: early larvae stages, L4: last larvae stage. Similar to the wild-type animals (Fig 5) DII delays the development of *unc-29* null mutant animals. Animal stage percentages are relative to the number of living animals at the indicated time point (n = 3).
(TIF)

S8 Fig DII larvicidal activity in *mutant strains*. L1 larvae of wild-type, *unc-38*, *unc-63*, *lev-1* and *lev-8* null mutants were exposed to DII (600 μM) and after 24, 48 and 72 h animal viability was evaluated. Data are presented as mean ± SEM. Statistical significance compared to the corresponding control at each time point (**p<0.01, ***p<0.001, ns: no statistically significant p>0.05; n = 3).
(TIF)

S1 Appendix Spectra for compounds 1–11. (PDF)

S1 Movie Non-treated egg hatching. (MP4)

S2 Movie DII-treated egg hatching. (MP4)
