# Supplementary figures and images for: Diisopropylphenyl-imidazole (DII): A new compound that exerts anthelmintic activity through novel molecular mechanisms
Source: PLoS Negl Trop Dis. 2018 Dec 17;12(12):e0007021. doi: 10.1371/journal.pntd.0007021 (PMC6312359; doi:10.1371/journal.pntd.0007021)

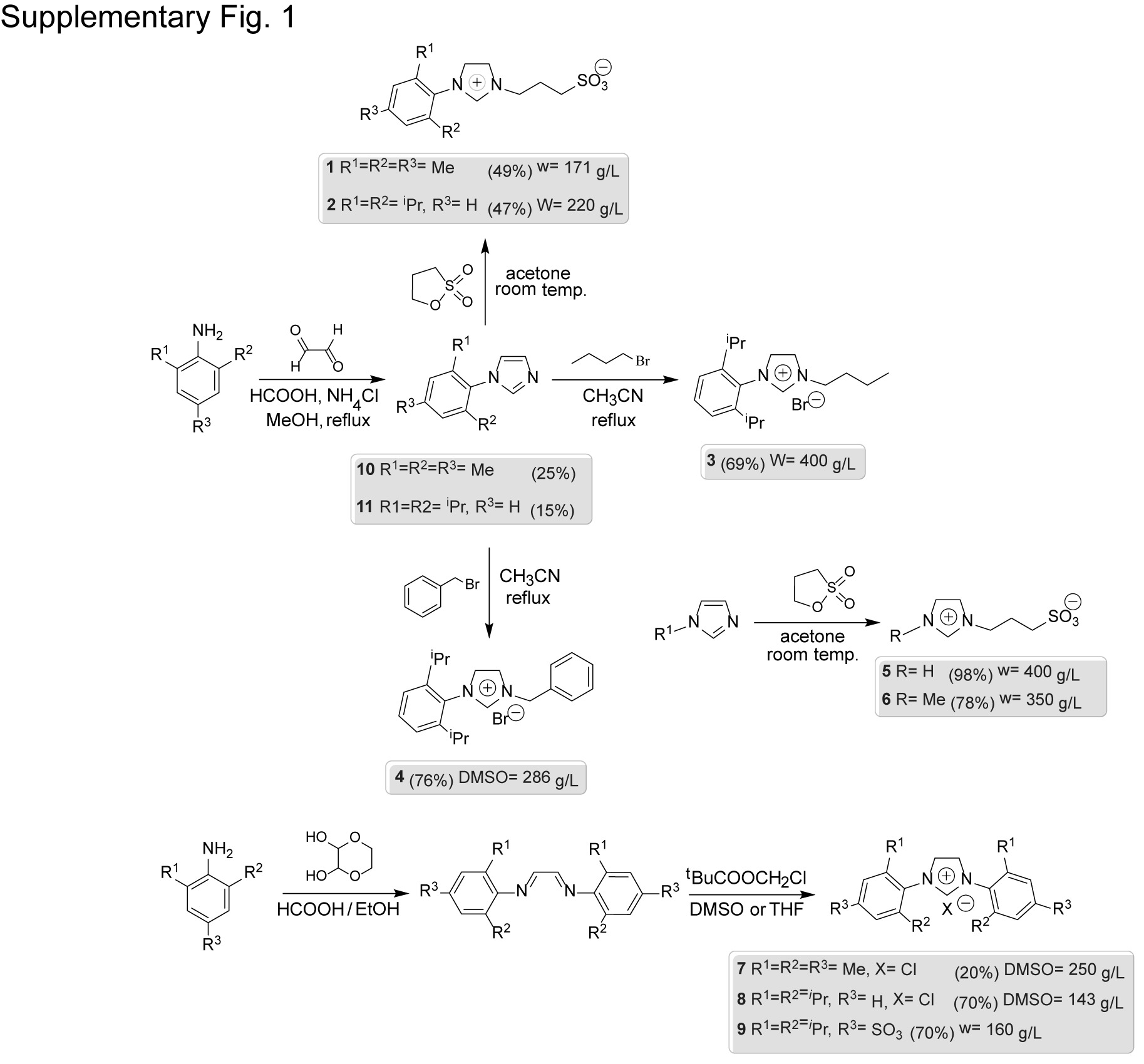

Supplement: S1 Fig — (TIF) [file pntd.0007021.s001.tif]

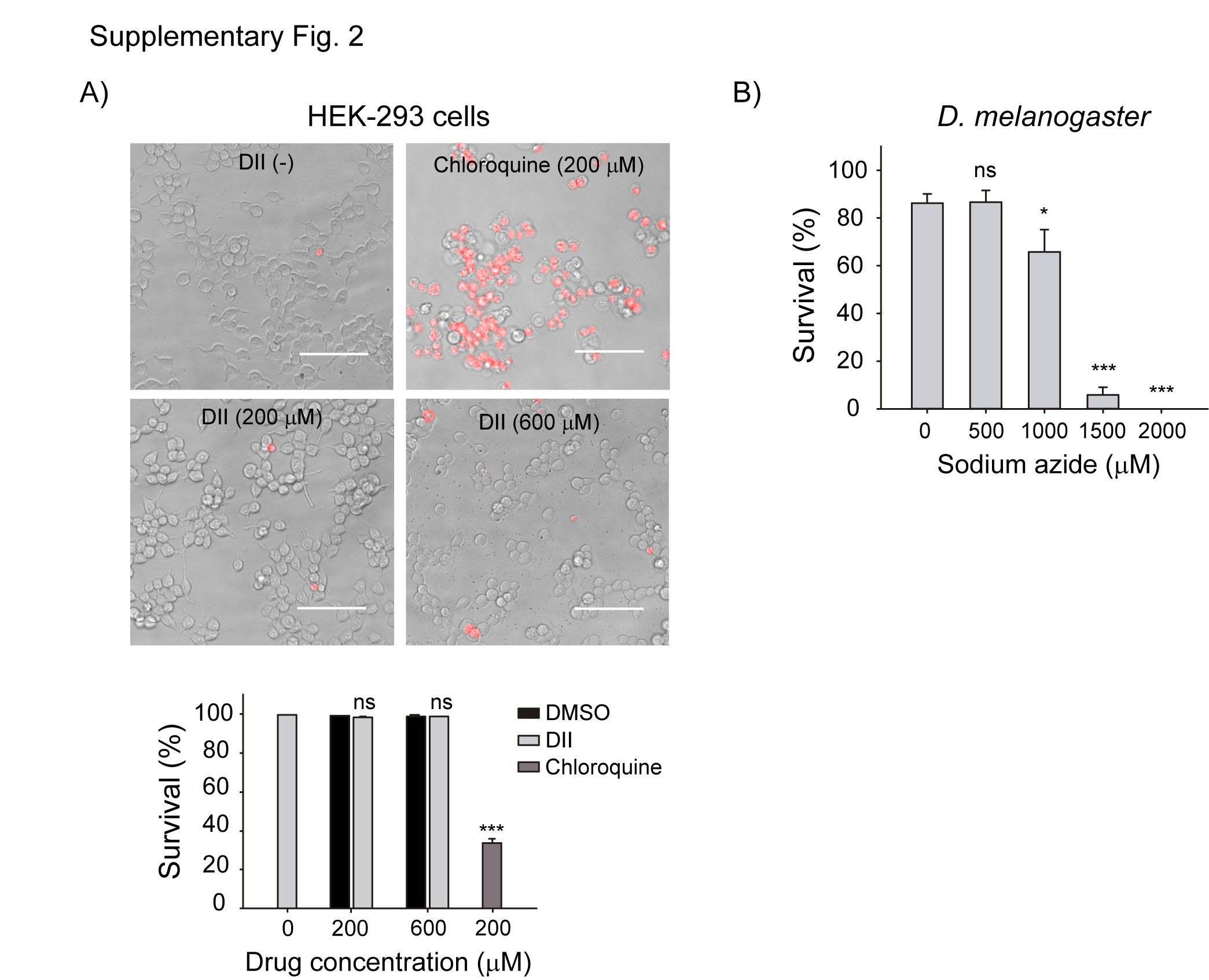

Supplement: S2 Fig — (A) DII effect on human cell cultures was evaluated using HEK-293 cells. Cells were exposed to DII (0–600 μM) and after 8 h of incubation, cell death was quantified using Propidium Iodide (PI) staining. Untreated and chloroquine-treated (200 μM) cells were used as a negative and positive control, respectively. Upper: PI staining microphotographs. Red staining accounts for dead cells. Bar scale: 100 μm. Bottom: Cell survival quantification. Results are presented as mean ± SEM (ns: no statistically significant, p > 0.05, ***p<0.001; n = 3) (B) Positive control for Drosophila larval viability assay. Fly larvae were exposed to Sodium Azide (0.5–2 mM) until they left the food to pupariate. Larval survival was calculated as the percentage of larvae that reached the pupal stage. Results are presented as mean ± SEM (ns: no statistically significant, p > 0.05, *p<0.05, ***p<0.001; n = 3). (TIF) [file pntd.0007021.s002.tif]

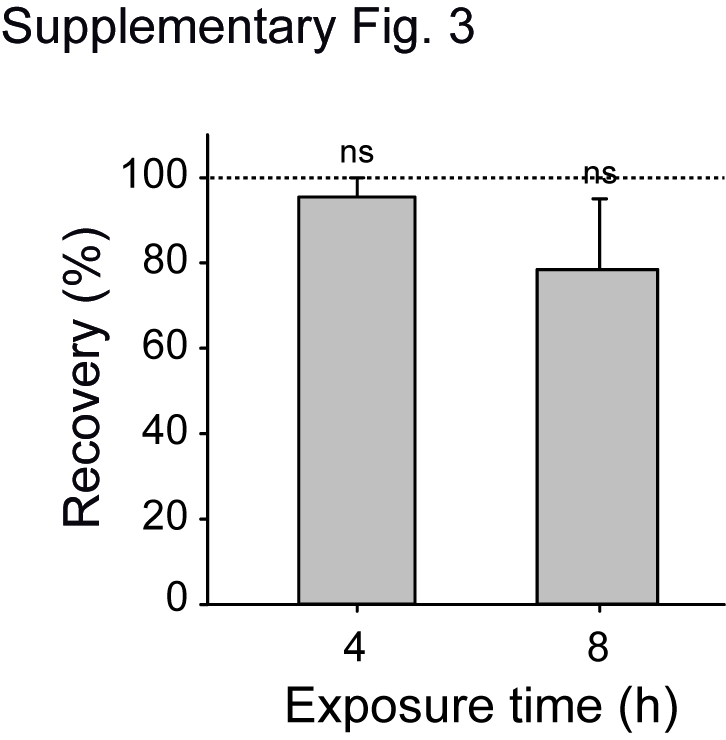

Supplement: S3 Fig — ~100 L4 worms were transferred to NGM plates containing DII (600 μM). After 4 and 8 h of exposure, survivors were transferred to regular NGM plates. Worm viability was scored 24 h later. Untreated worms were considered as 100% of viability (discontinued line). Results are presented as mean ± SEM. Statistical significance compared to untreated worms (ns: no statistically significant, p > 0.05; n = 3). (TIF) [file pntd.0007021.s003.tif]

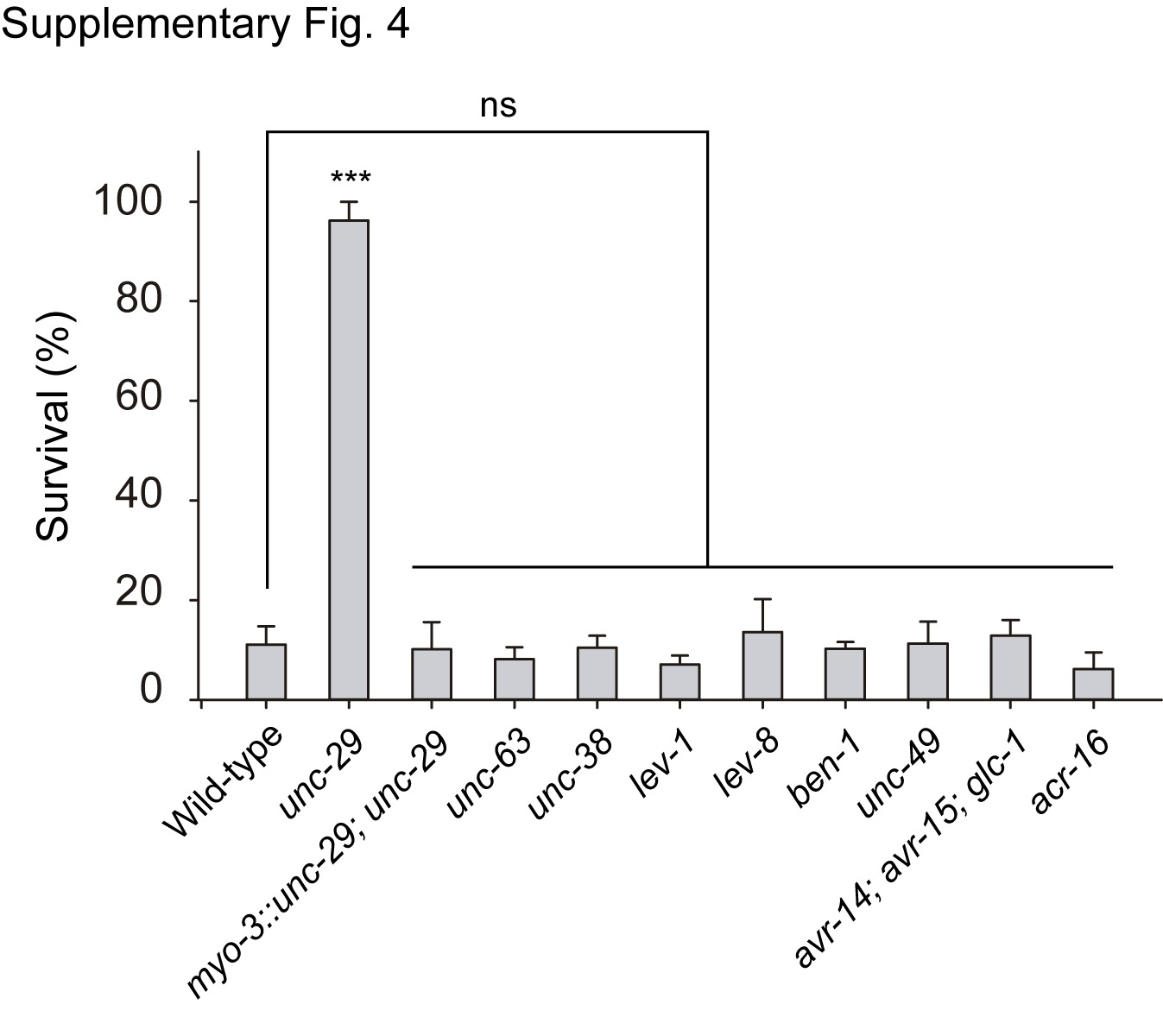

Supplement: S4 Fig — ~40 L4 mutant animals were exposed to DII (50 μM) for 96 h. Worm survival was subsequently scored. Only unc-29 null mutant strain was resistant to DII anthelmintic activity.‬‬‬ Results are presented as mean ± SEM. Statistical significance compared to wild-type worms (ns: no statistically significant, ***p<0.001; n = 3). (TIF) [file pntd.0007021.s004.tif]

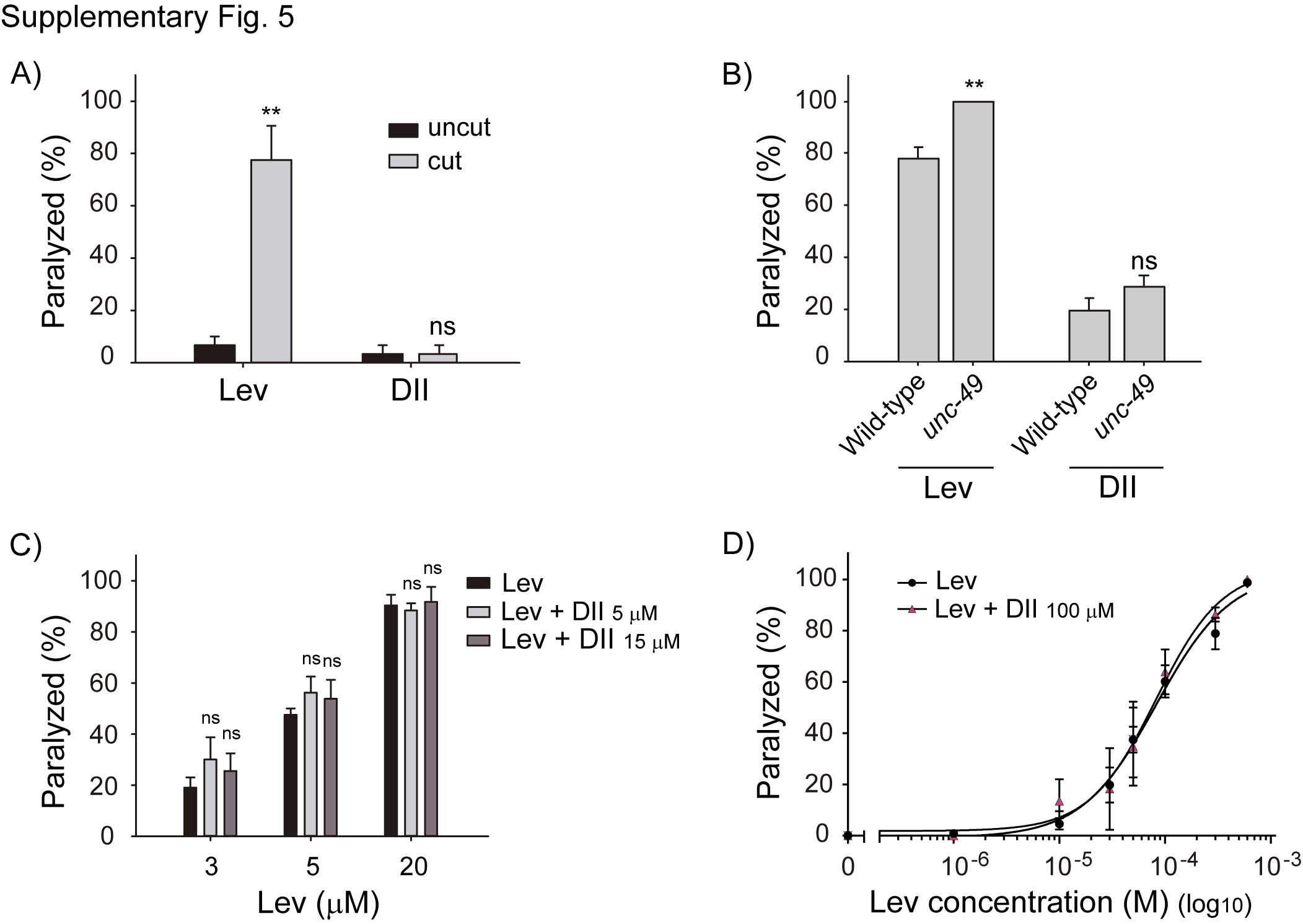

Supplement: S5 Fig — (A) Acute paralysis assays. Paralysis was scored in intact and cut adult worms (see Methods) exposed to levamisole (10 μM) or DII (600 μM) for 10 minutes. DII is unable to induce paralysis even in cut animals. Results are presented as mean ± SEM. Statistical significance compared to uncut worms (ns: no statistically significant, **p<0.01; n = 3). (B) Acute paralysis assays on CB407 unc-49(e407)III mutant strain. 30–40 young adult worms were exposed to each drug (100 μM levamisole or 100 μM DII). After 10 minutes, the number of paralyzed animals was scored. As expected CB407 strain is hypersensitive to levamisole. No significant differences between wild-type animals and CB407 strains were observed in DII-treated animals. Data are presented as mean ± SEM. Statistical significance compared to wild-type worms (ns: no statistically significant p>0.05 **p<0.01; n = 4). (C) Effect of DII on levamisole-induced paralysis in cut animals. Cut adult worms were exposed to levamisole (3, 5, 20 μM) alone and in the presence of two different concentrations of DII (5 and 15 μM). Similar to the observations in intact animals, the presence of DII does not impair levamisole action. Data are presented as mean ± SEM. Statistical significance compared to levamisole (Lev) treated worms. (ns: no statistically significant p>0.05; n = 3) (D) Levamisole dose-response curves after long pre-exposure to DII. Animals were exposed to DII (100 μM) for 24 h. After this treatment, they were exposed to a range of levamisole concentrations (1–600 μM) in the presence of DII (100 μM). Control curves were performed similarly, without the addition of DII neither in the preincubation nor in the paralysis assay. Data are fitted with a 4PL curve. (Black circle) EC50 = 82.43 ± 1.23 μM, Hill slope = 1.23 ± 0.26, R2 = 0.95. (Pink triangle) EC50 = 82.22 ± 1.39 μM, Hill slope = 1.38 ± 0.54, R2 = 0.88. Each concentration point represents the mean value ± SEM of three independent experiments. No differences were obse [file pntd.0007021.s005.tif]

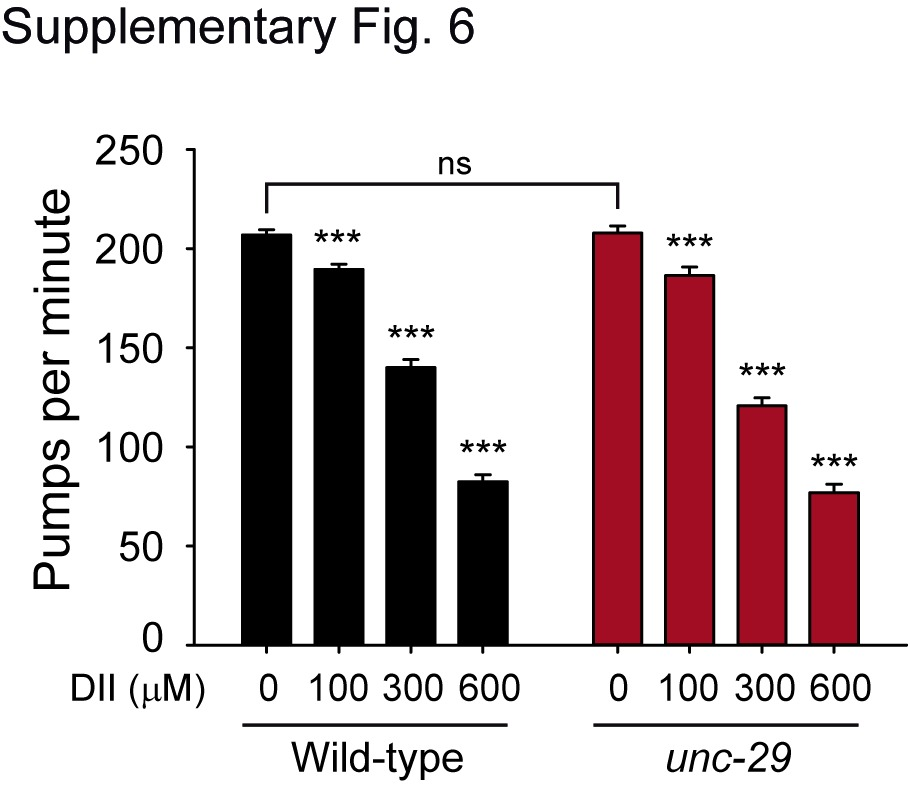

Supplement: S6 Fig — Wild-type and unc-29 young adult worms (24 h past L4) were transferred to bacteria-seeded NGM plates containing different DII concentrations. After two hours of DII exposure, the number of contractions in the terminal bulb of the pharynx (pumps per minute) was counted using a stereomicroscope at 50x magnification. DII inhibits the pharyngeal pumping. However, the fact that this inhibition also occurs in unc-29 mutants suggests that the effect on pumping rate does not underlie DII nematicidal action. Bars represent the mean ± SEM from n = 20 animals per condition. Statistical differences compared to the non-treated condition (0 μM DII) within the same strain (ns: no statistically significant p>0.05, ***p<0.001). (TIF) [file pntd.0007021.s006.tif]

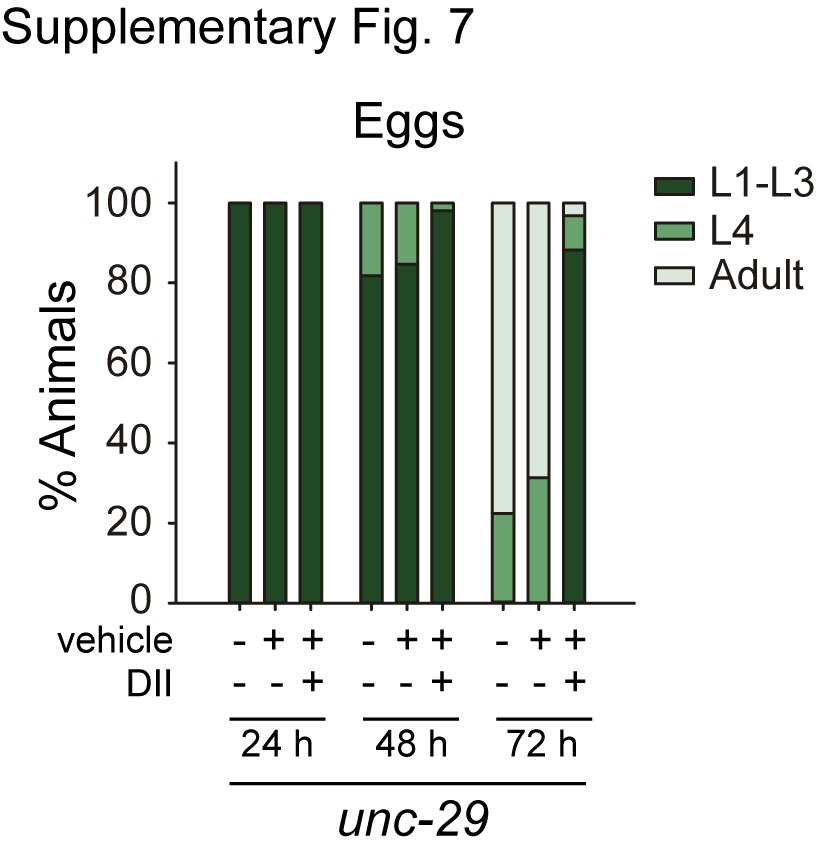

Supplement: S7 Fig — Isolated eggs were exposed to DII (600 μM) and at each time point (24, 48 and 72 h), animal developmental stages were scored. L1-L3: early larvae stages, L4: last larvae stage. Similar to the wild-type animals (Fig 5) DII delays the development of unc-29 null mutant animals. Animal stage percentages are relative to the number of living animals at the indicated time point (n = 3). (TIF) [file pntd.0007021.s007.tif]

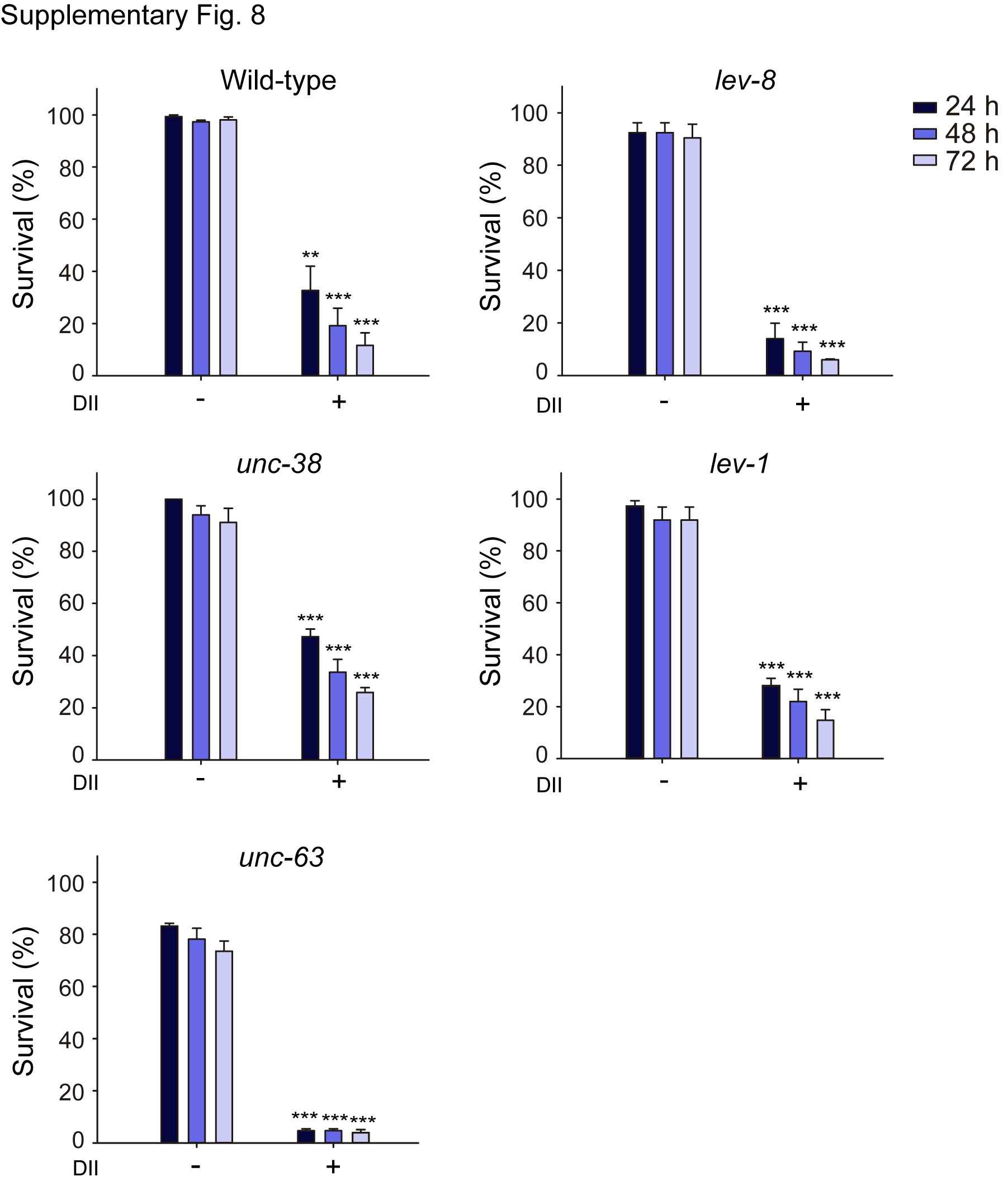

Supplement: S8 Fig — L1 larvae of wild-type, unc-38, unc-63, lev-1 and lev-8 null mutants were exposed to DII (600 μM) and after 24, 48 and 72 h animal viability was evaluated. Data are presented as mean ± SEM. Statistical significance compared to the corresponding control at each time point (**p<0.01, ***p<0.001, ns: no statistically significant p>0.05; n = 3). (TIF) [file pntd.0007021.s008.tif]
