# Supplementary material for: Diisopropylphenyl-imidazole (DII): A new compound that exerts anthelmintic activity through novel molecular mechanisms
Source: PLoS Negl Trop Dis. 2018 Dec 17;12(12):e0007021. doi: 10.1371/journal.pntd.0007021 (PMC6312359; doi:10.1371/journal.pntd.0007021)

Diisopropylphenyl-imidazole: A new compound that exerts anthelmintic activity through novel molecular mechanisms

Gabriela Blanco[1,2], María Soledad Vela Gurovic[2,3], Gustavo Fabián Silbestri[4], Andrés Garelli[1,2], Sebastián Giunti[1,2], Diego Rayes[1,2]* and María José De Rosa[1,2]*.

1- Instituto de Investigaciones Bioquímicas de Bahía Blanca (INIBIBB) CCT UNS-CONICET. Bahía Blanca, Argentina.
2- Dpto de Biología, Bioquímica y Farmacia, Universidad Nacional del Sur. Bahía Blanca, Argentina.
3- CERZOS UNS-CONICET CCT. Bahía Blanca, Argentina.
4- Dpto de Química, Universidad Nacional del Sur (UNS)-CONICET. Instituto de Química del Sur (INQUISUR), Bahía Blanca, Argentina.

Supplementary Material

Table of Contents

Title page and detailed list of contents of the Supporting Information (SI)	1
^{1}H and ^{13}C NMR of 1-Mesithyl-3-(3-sulfonatopropyl)imidazolium (**1**)	2
^{1}H and ^{13}C NMR of 1-(2,6-Diisopropylphenyl)-3-(3-sulfonatopropyl)imidazolium (**2**)	3
^{1}H and ^{13}C NMR of 1-(2,6-Diisopropylphenyl)-3-(propyl)imidazolium bromide (**3**)	4
^{1}H and ^{13}C NMR of 1-(2,6-Diisopropylphenyl)-3-(propyl)imidazolium bromide (**4**)	5
^{1}H and ^{13}C NMR of 1-(3-sulfonatopropyl)imidazolium (**5**)	6
^{1}H and ^{13}C NMR of 1-Methyl-3-(3-sulfonatopropyl)imidazolium (**6**)	8
^{1}H and ^{13}C NMR of 1,3-bis(Mesithyl)imidazolium Chloride (**7**)	9
^{1}H and ^{13}C NMR of 1,3-bis(2,6-diisopropyl-phenyl)imidazolium Chloride (**8**)	10
^{1}H and ^{13}C NMR of 1,3-bis(2,6-diisopropyl-4sodiumsulfonatophenyl)imidazolium (**9**)	11
^{1}H and ^{13}C NMR of Mesityl imidazol (**10**)	12
^{1}H and ^{13}C NMR of Diisopropyl imidazol (**11**)	13

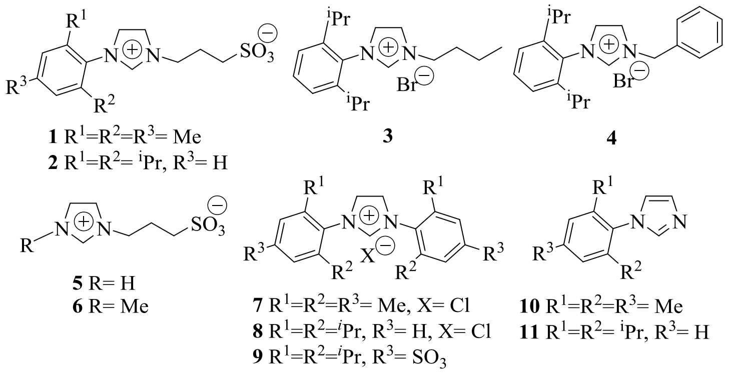

Imidazoliun salts and neutral compounds synthetized.

1-Mesithyl-3-(3-sulfonatopropyl)imidazolium (**1**) (^{1}H-NMR, DMSO-D$_6$)

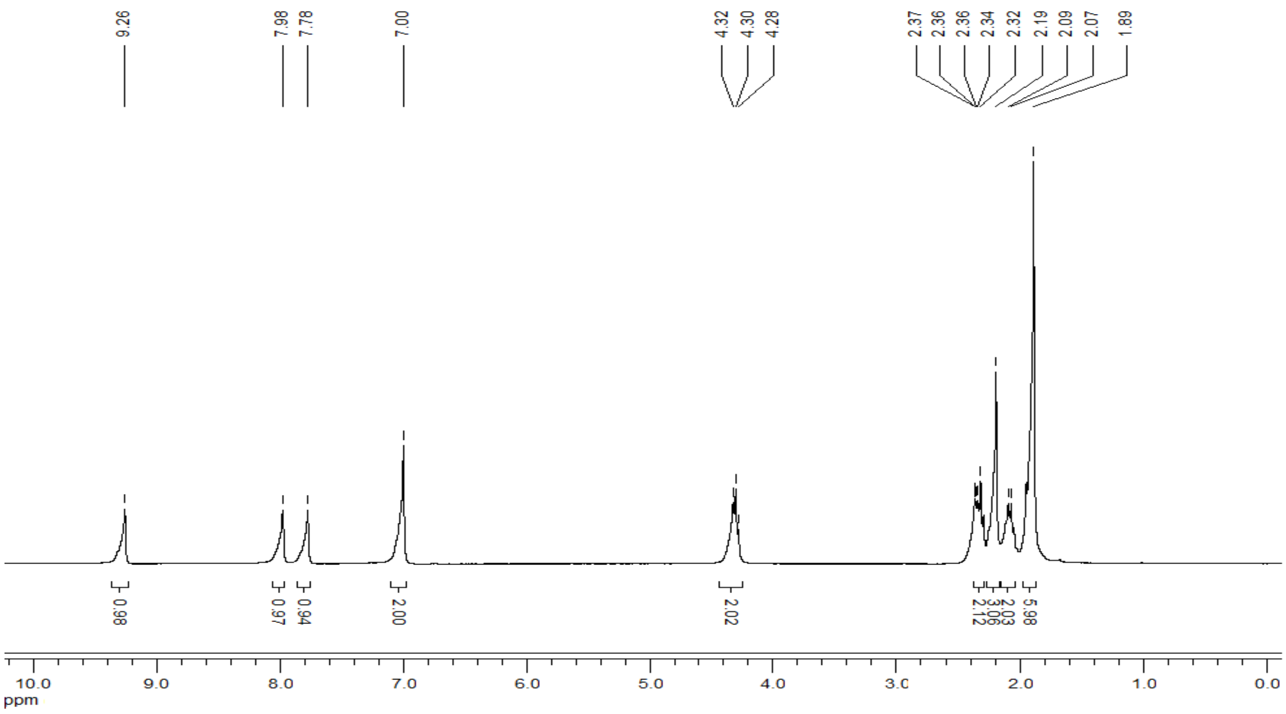

1-Mesithyl-3-(3-sulfonatopropyl)imidazolium (**1**) (^{13}C-NMR, DMSO-d$_6$)

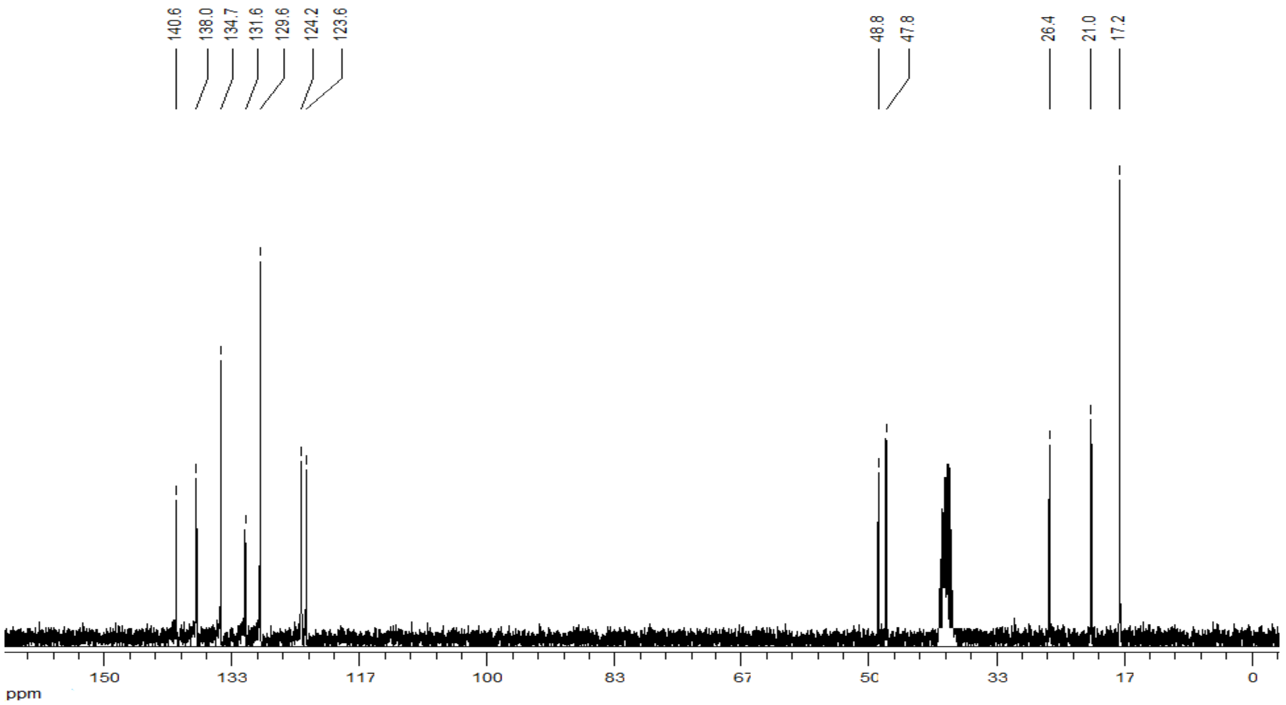

1-(2,6-Diisopropylphenyl)-3-(3-sulfonatopropyl)imidazolium (**2**) (^{1}H-NMR, D_2O)

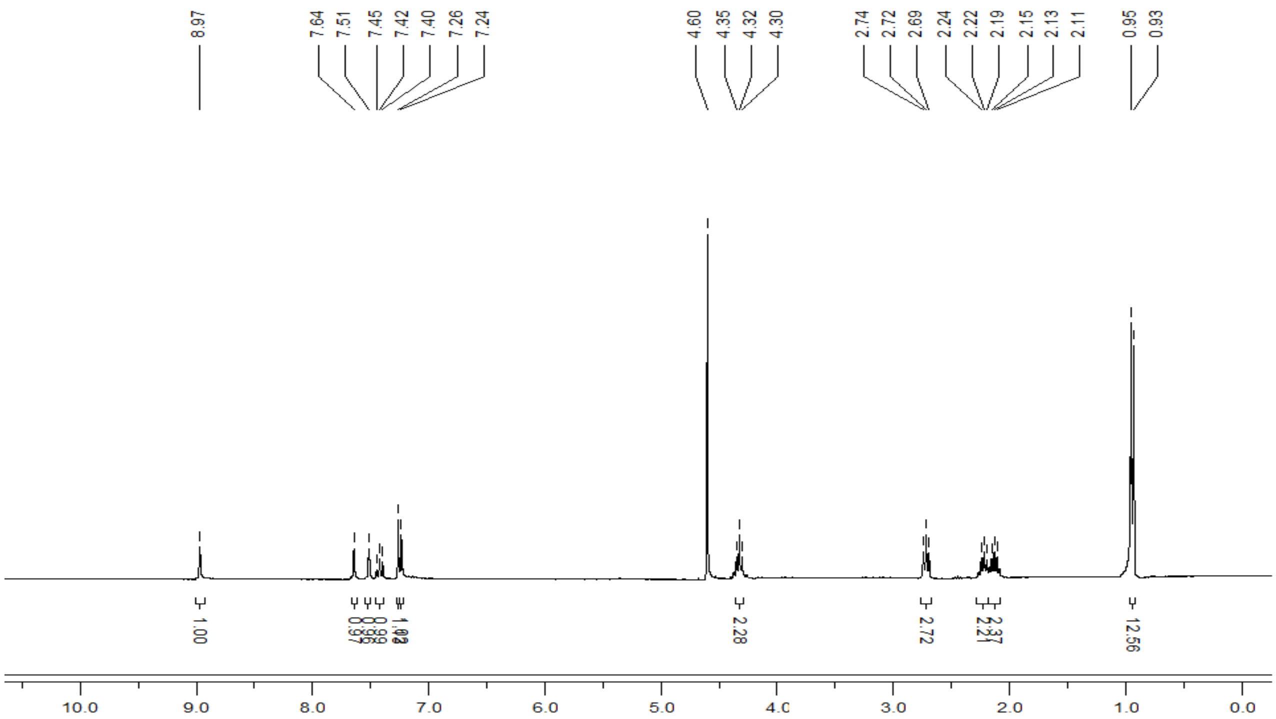

1-(2,6-Diisopropylphenyl)-3-(3-sulfonatopropyl)imidazolium (**2**) (^{13}C-NMR, D_2O)

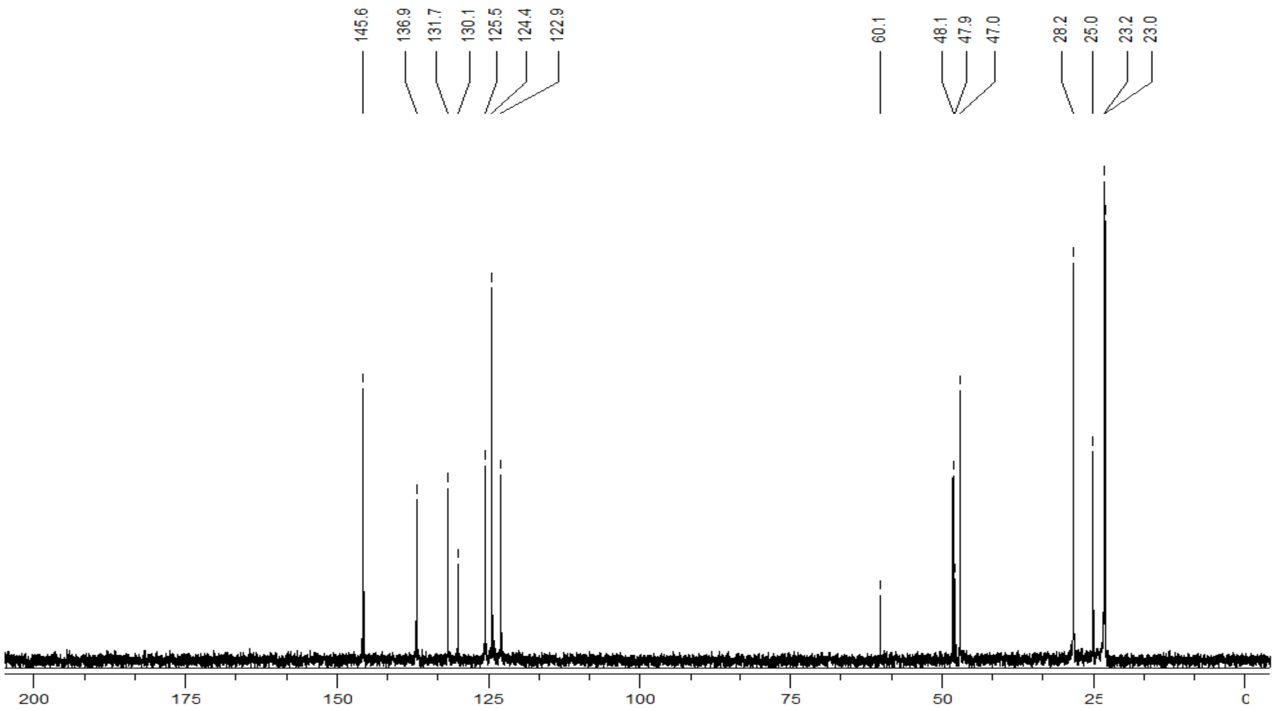

1-(2,6-Diisopropylphenyl)-3-(propyl)imidazolium bromide (**3**) (^{1}H-NMR, $CDCl_3$)

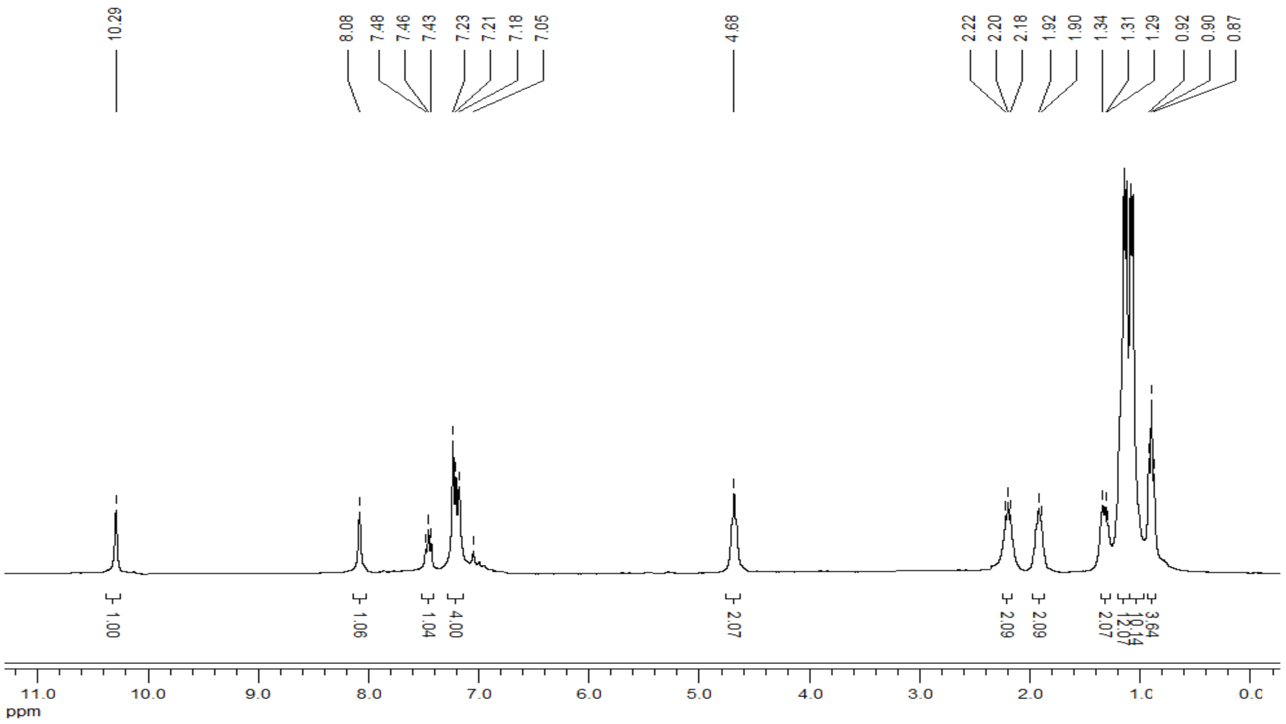

1-(2,6-Diisopropylphenyl)-3-(propyl)imidazolium bromide (**3**) (^{13}C-NMR, $CDCl_3$)

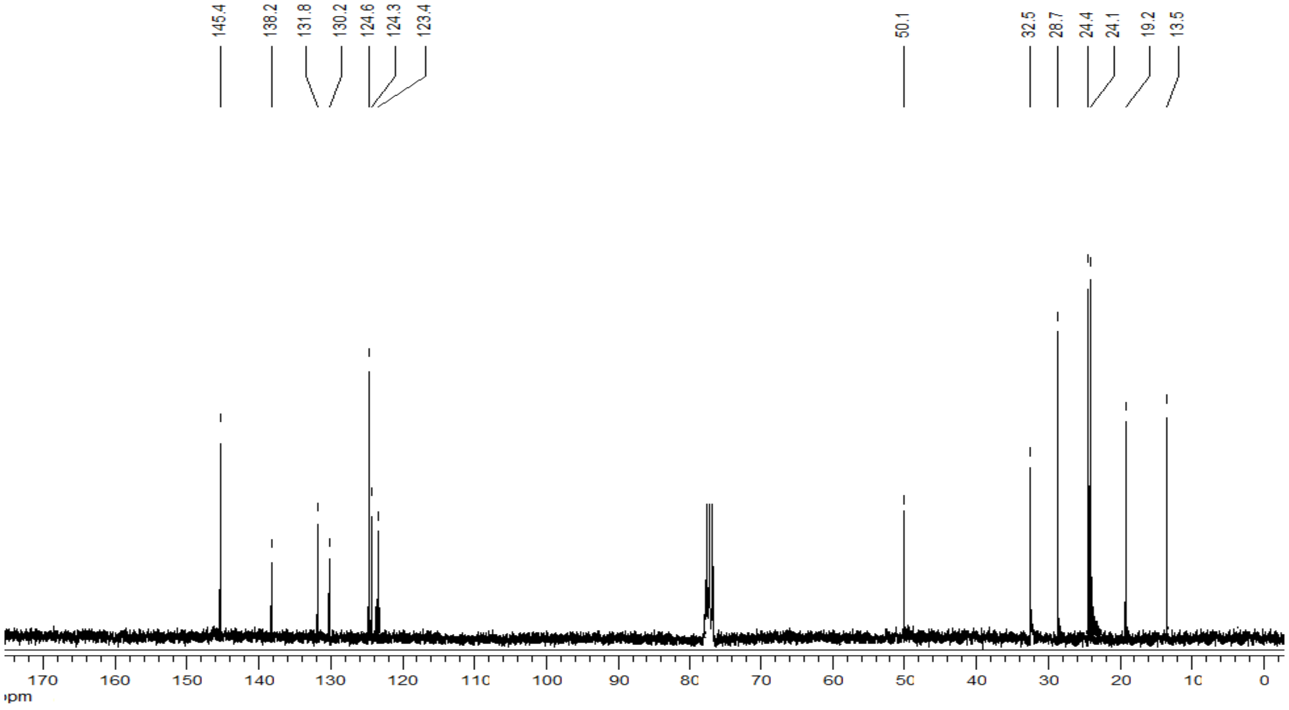

1-(2,6-Diisopropylphenyl)-3-(benzyl)imidazolium bromide (**4**) (^{1}H-NMR, $CDCl_3$)

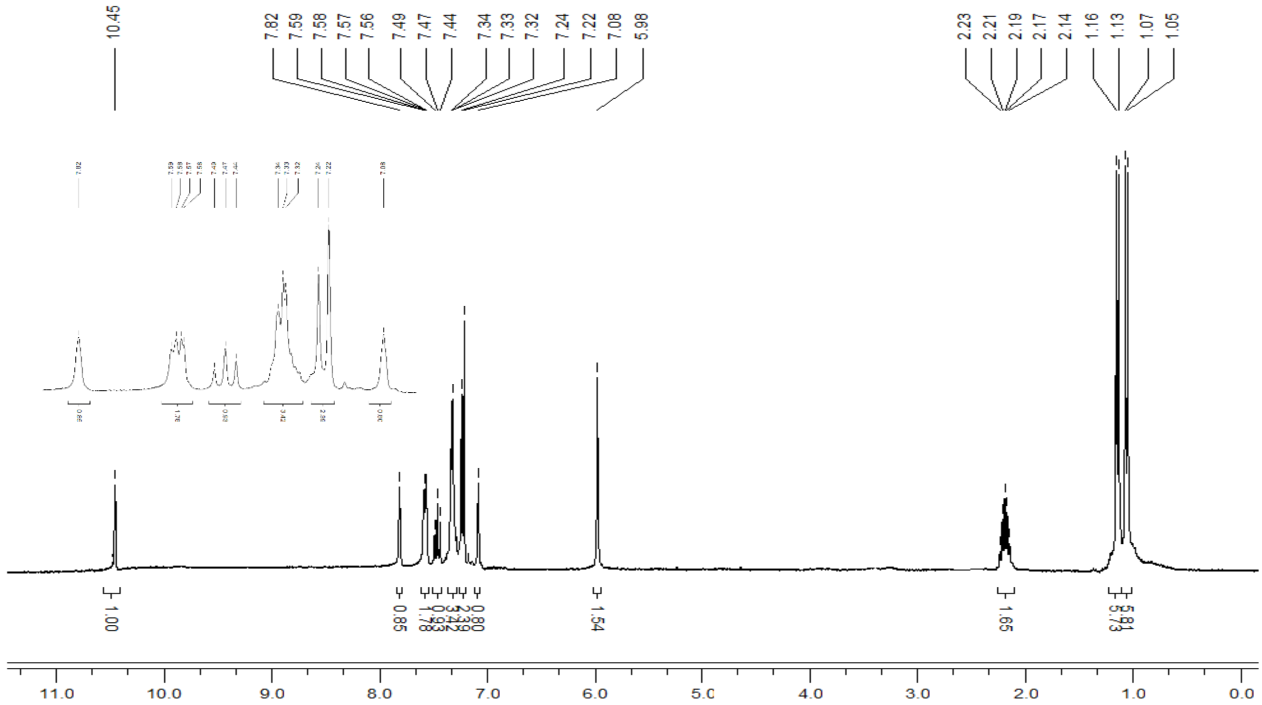

1-(2,6-Diisopropylphenyl)-3-(benzyl)imidazolium bromide (**4**) (^{13}C-NMR, $CDCl_3$)

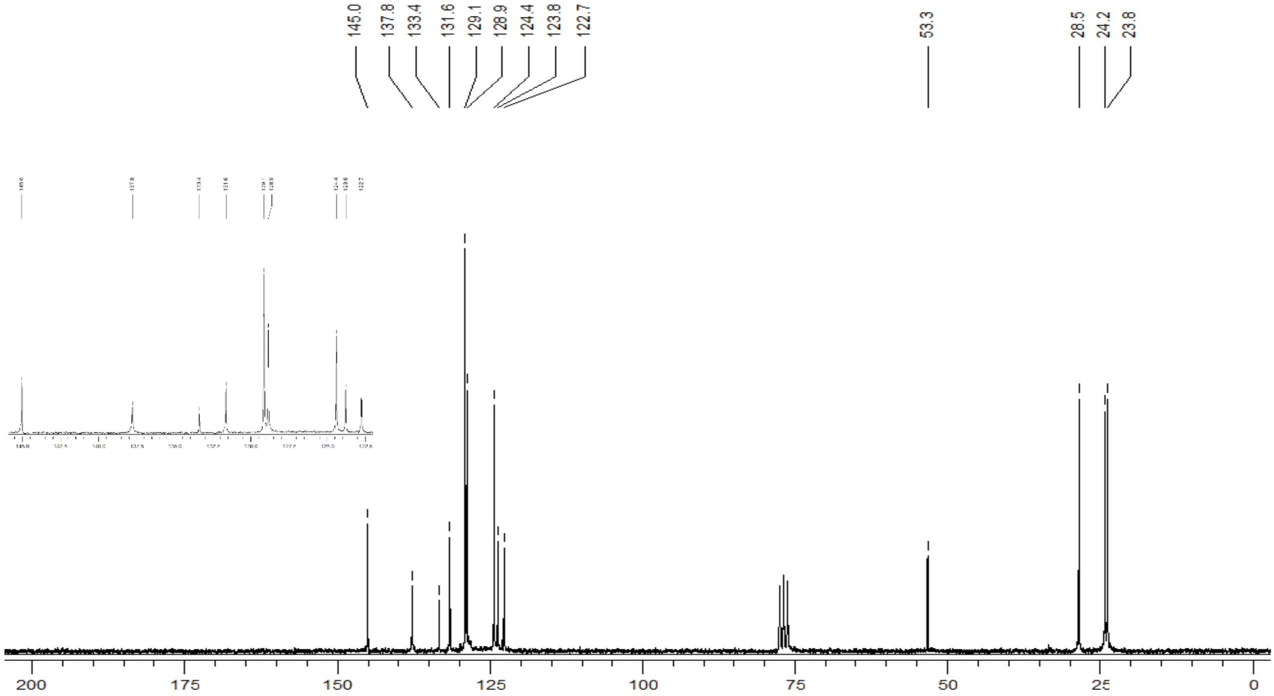

1-(3-sulfonatopropyl)imidazolium (**5**) (^{1}H-NMR, D_2O)

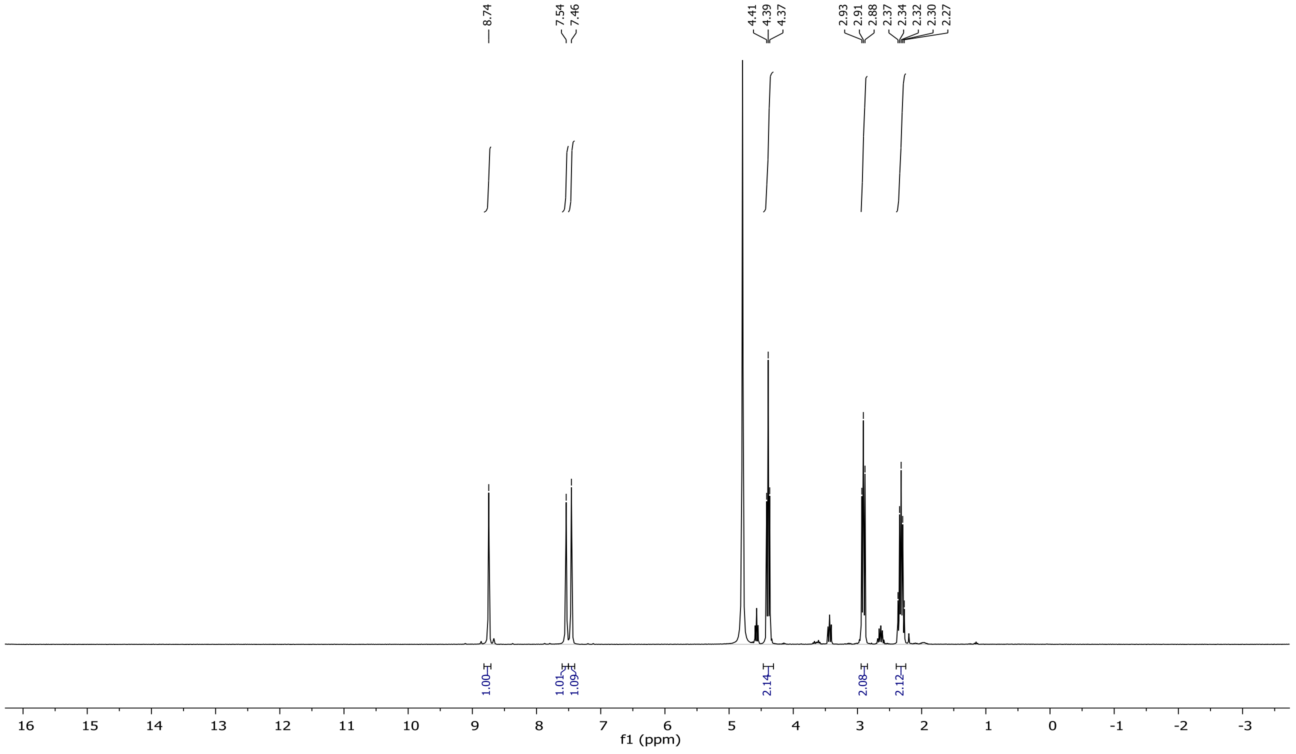

1-(3-sulfonatopropyl)imidazolium (**5**) (^{13}C-NMR, D_2O)

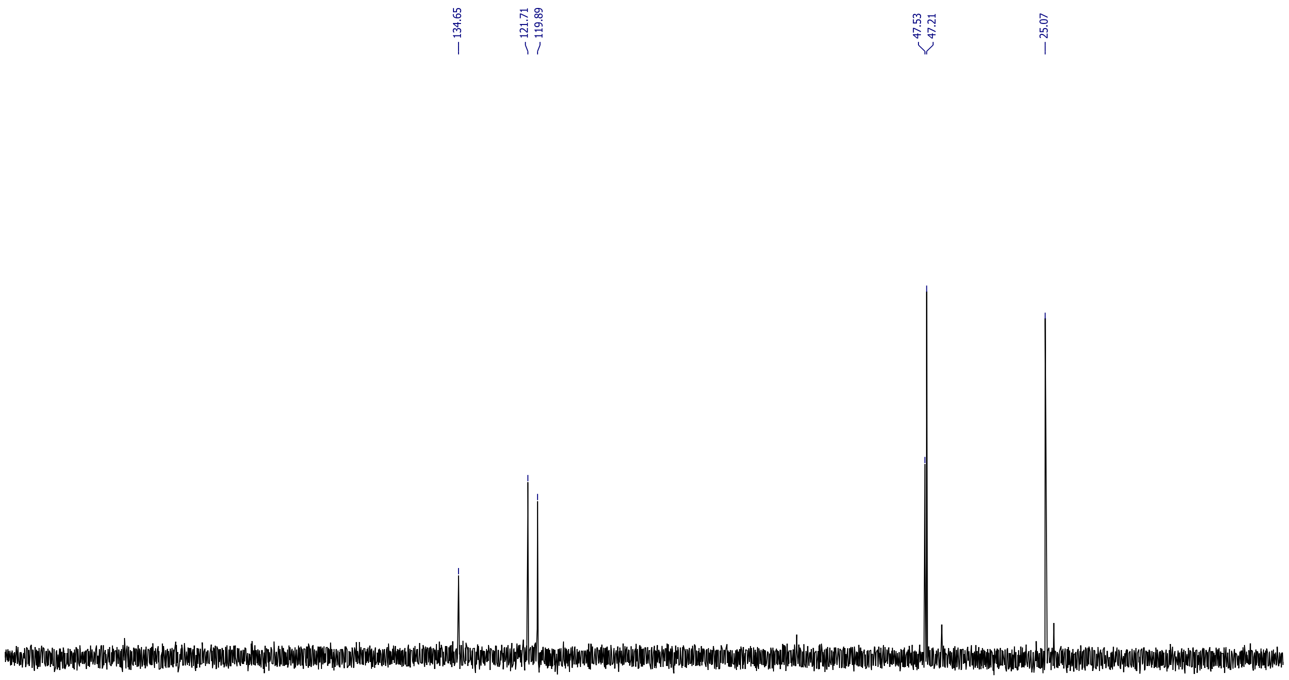

1-(3-sulfonatopropyl)imidazolium (**5**) (^{1}H-NMR, DMSO-d_6)

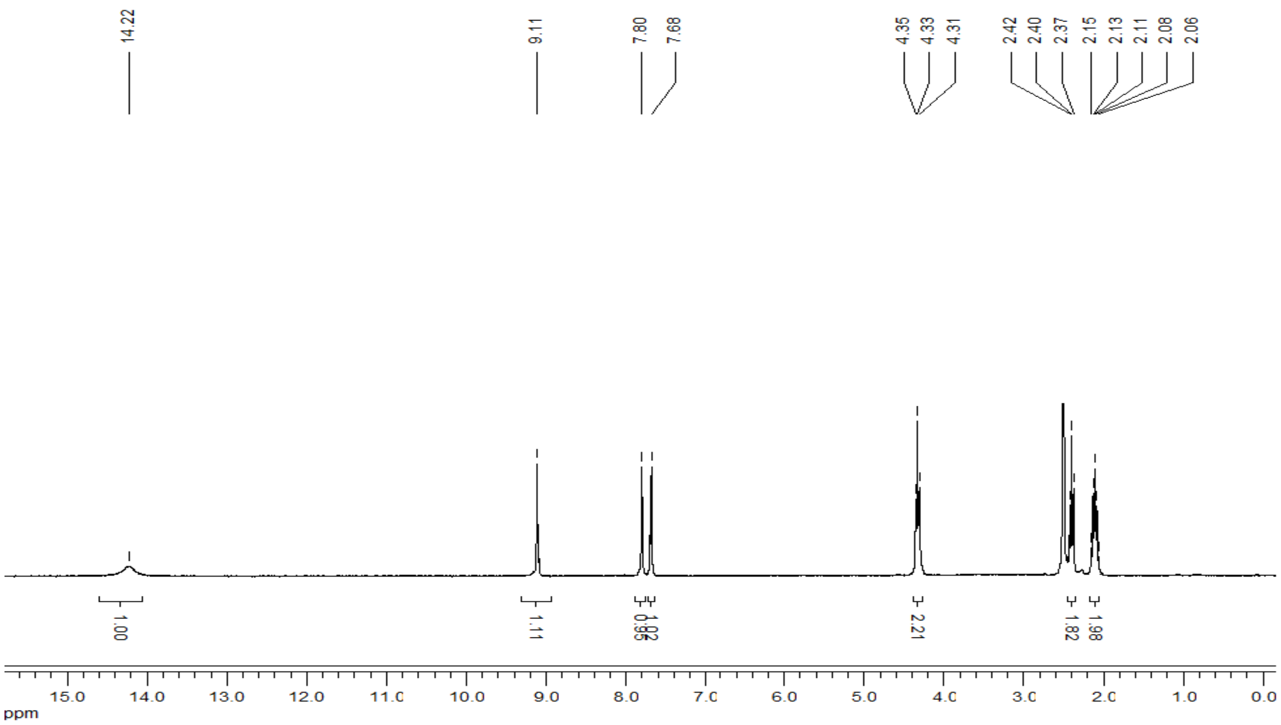

1-(3-sulfonatopropyl)imidazolium (**5**) (^{13}C-NMR, DMSO-d_6)

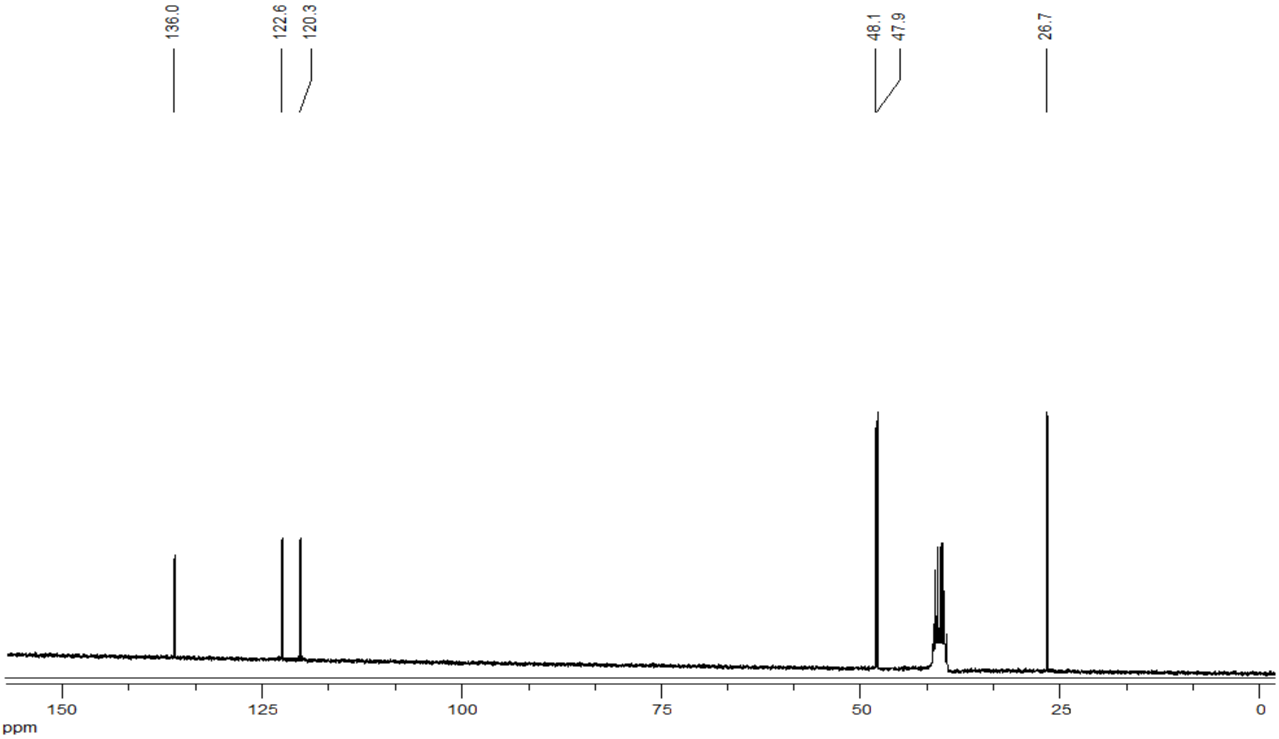

1-Methyl-3-(3-sulfonatopropyl)imidazolium (**6**) (^{1}H-NMR, D_2O)

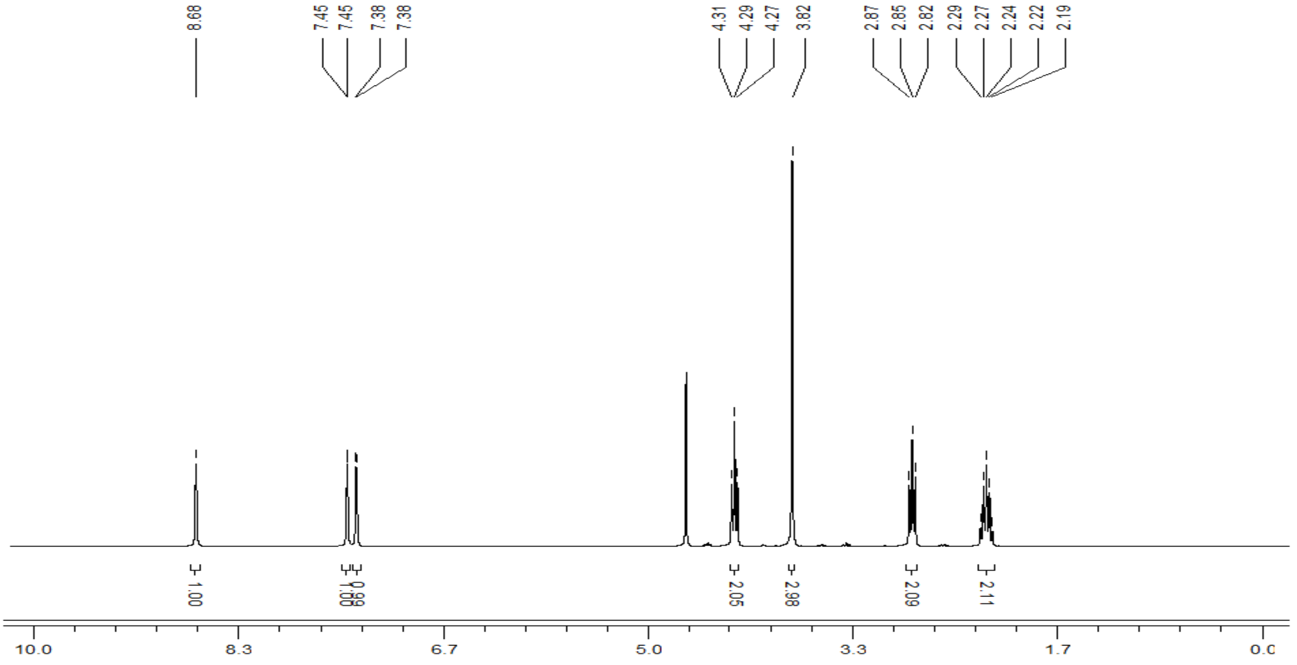

1-Methyl-3-(3-sulfonatopropyl)imidazolium (**6**)

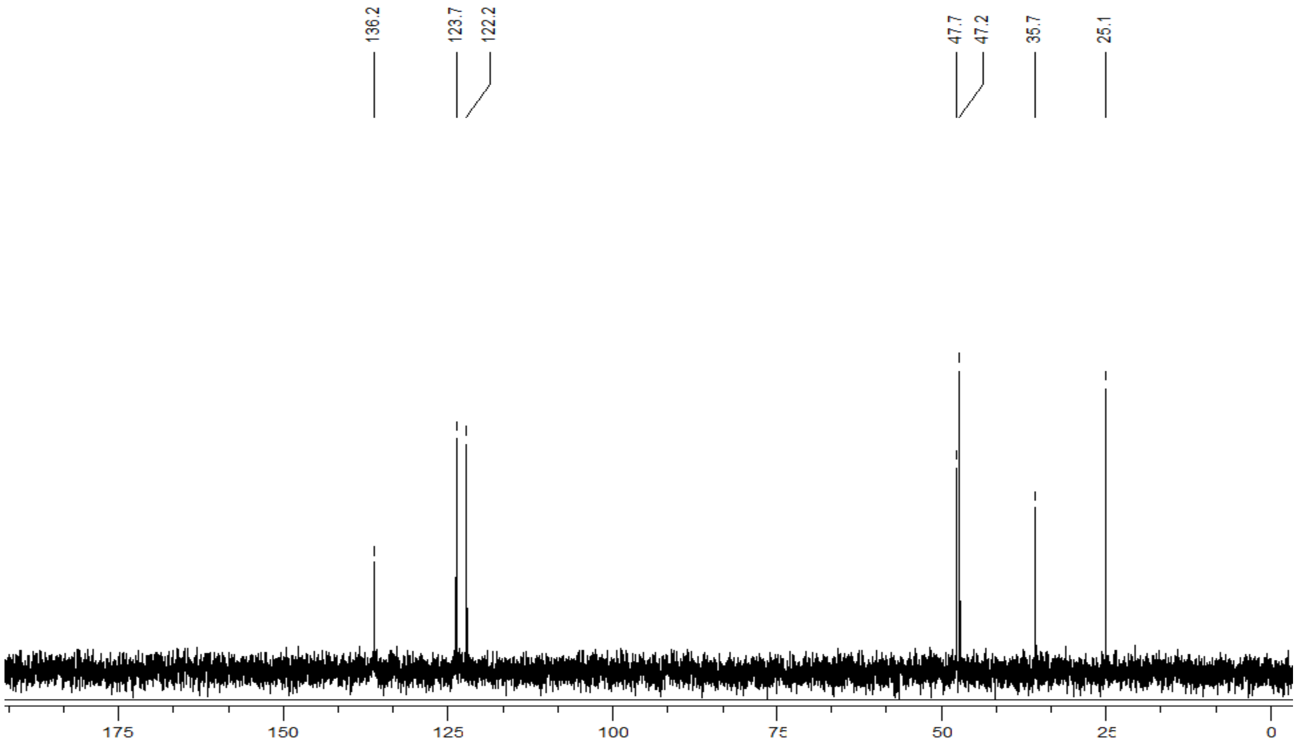

1,3-bis(Mesithyl)imidazolium Chloride (**7**) (^{1}H-NMR, $CDCl_3$)

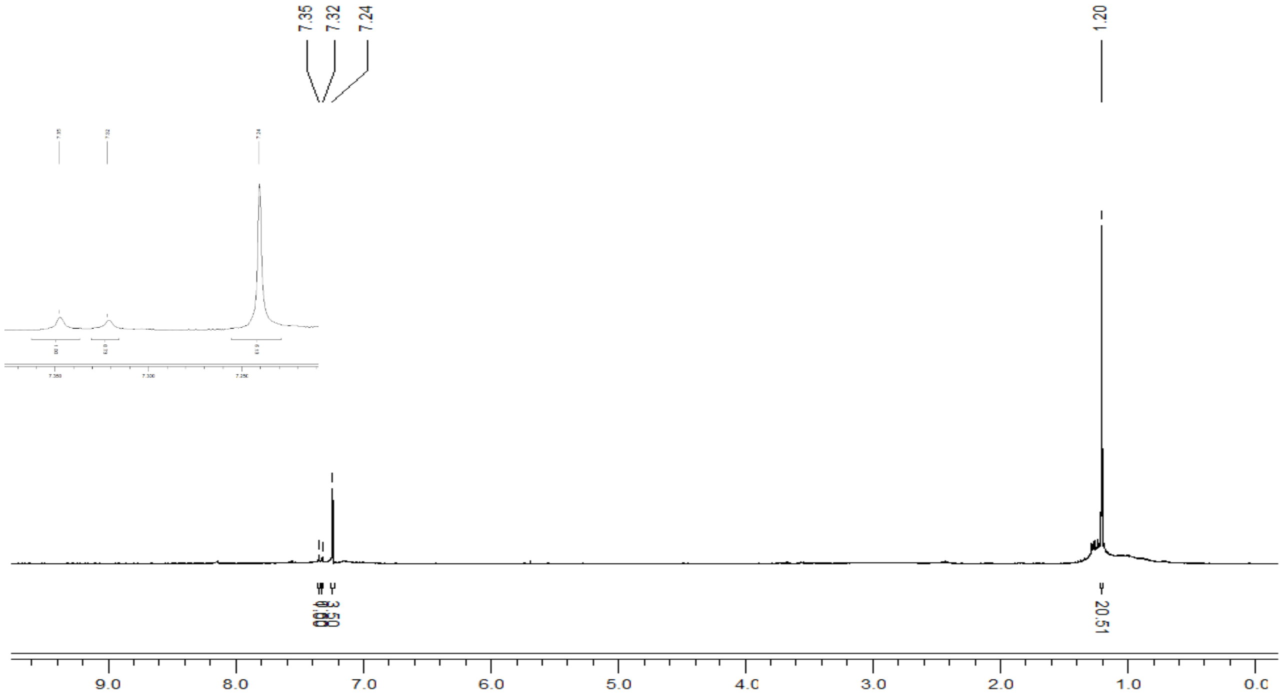

1,3-bis(Mesithyl)imidazolium Chloride (**7**) (^{13}C-NMR, $CDCl_3$)

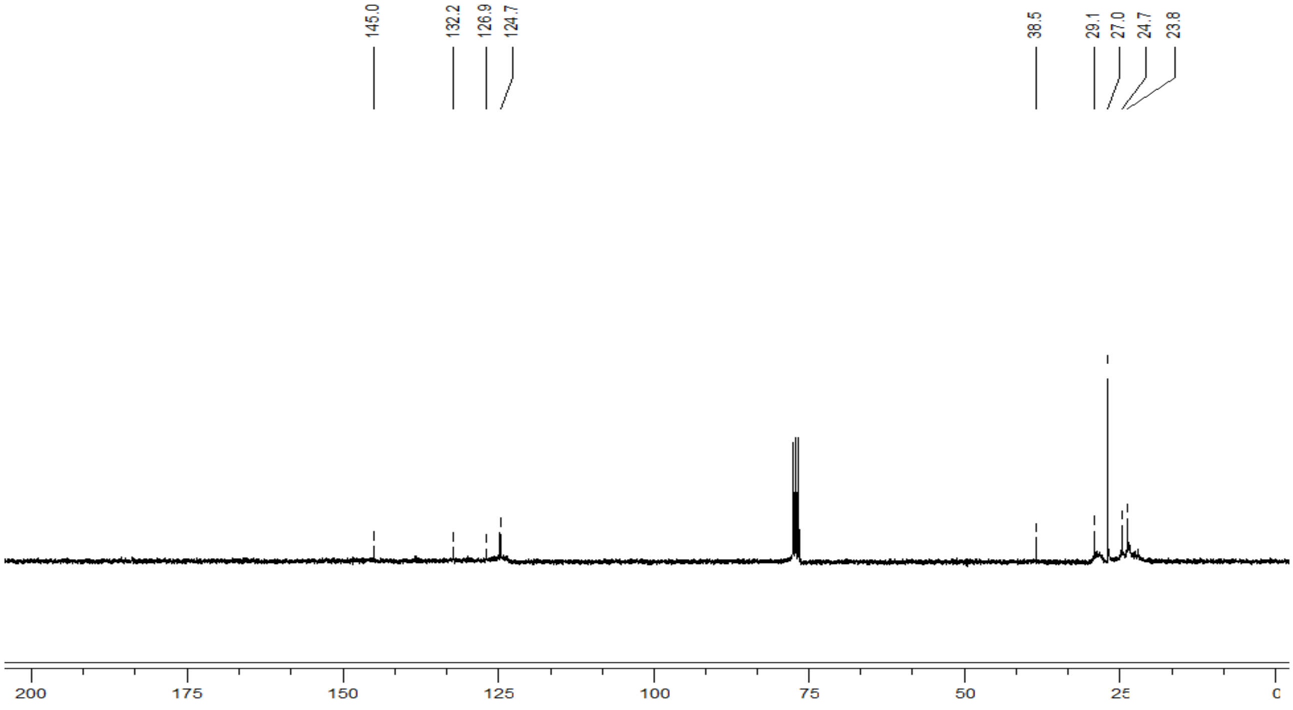

1,3-bis(2,6-diisopropyl-phenyl)imidazolium Chloride (**8**) (^{1}H-NMR, $CDCl_3$)

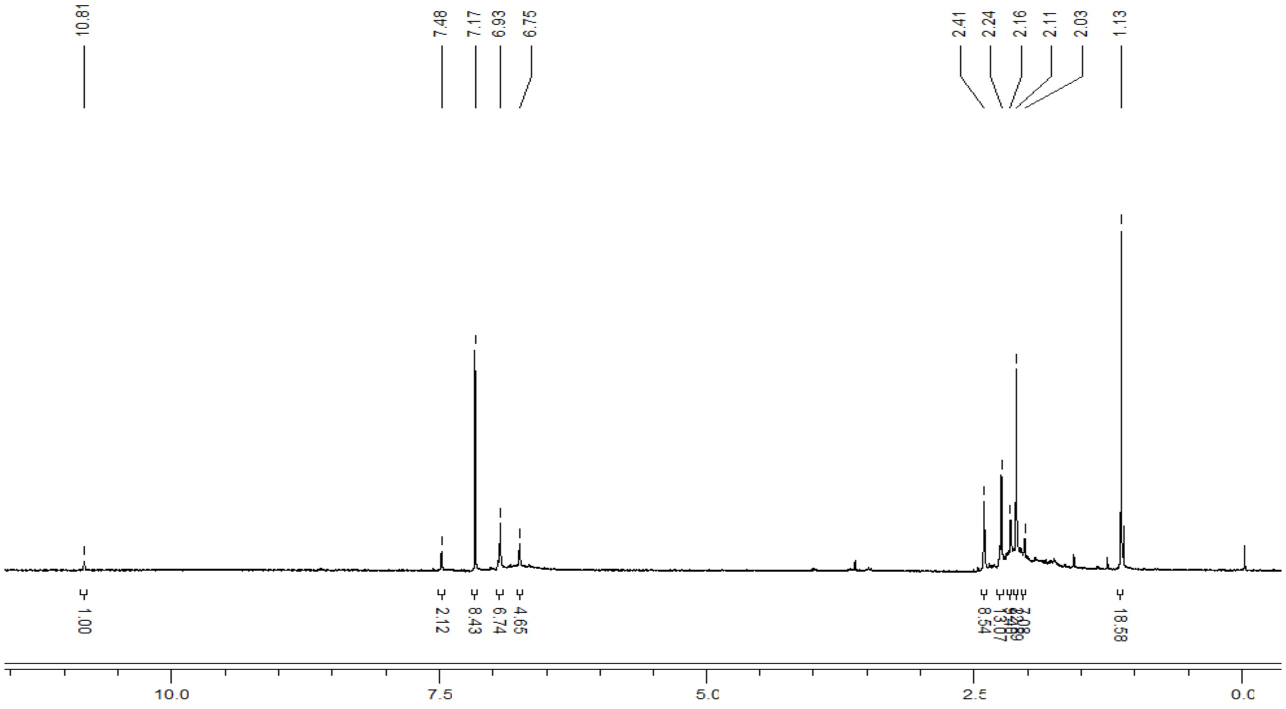

1,3-bis(2,6-diisopropyl-phenyl)imidazolium Chloride (**8**) (^{13}C-NMR, $CDCl_3$)

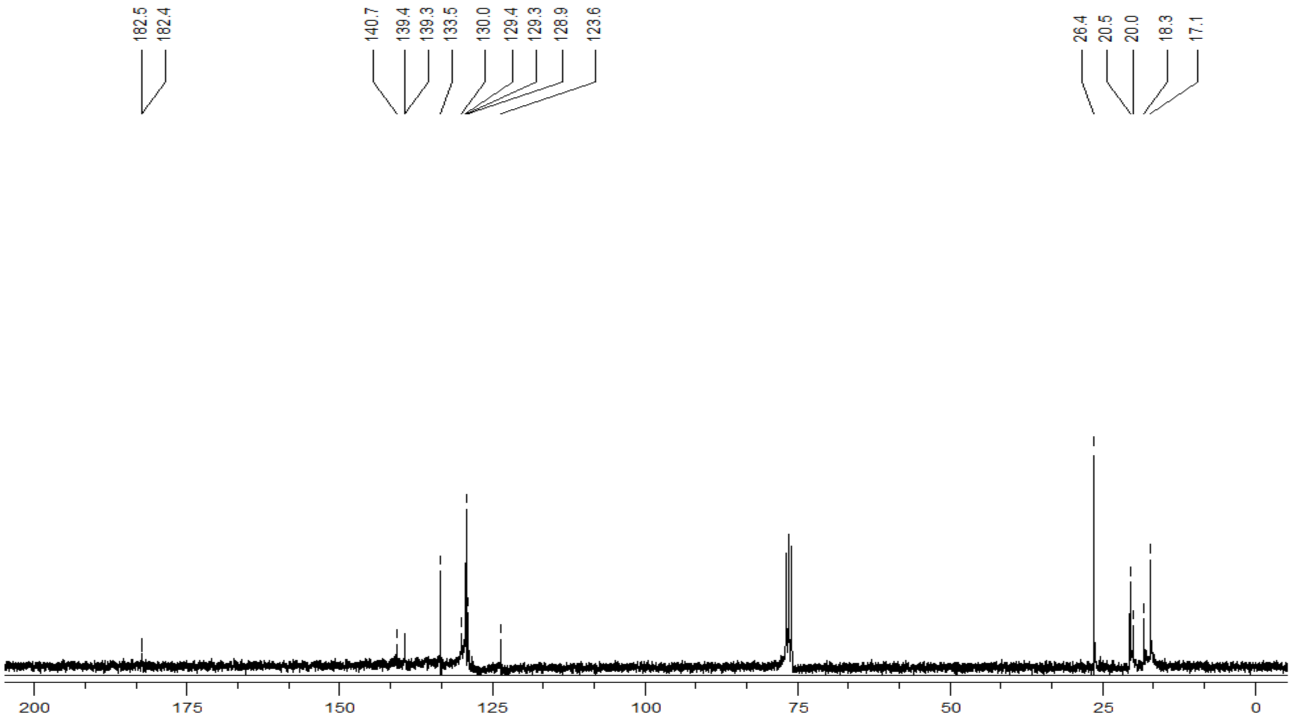

1,3-bis(2,6-diisopropyl-4sodiumsulfonatophenyl)imidazolium (**9**) (^{1}H-NMR, D_2O)

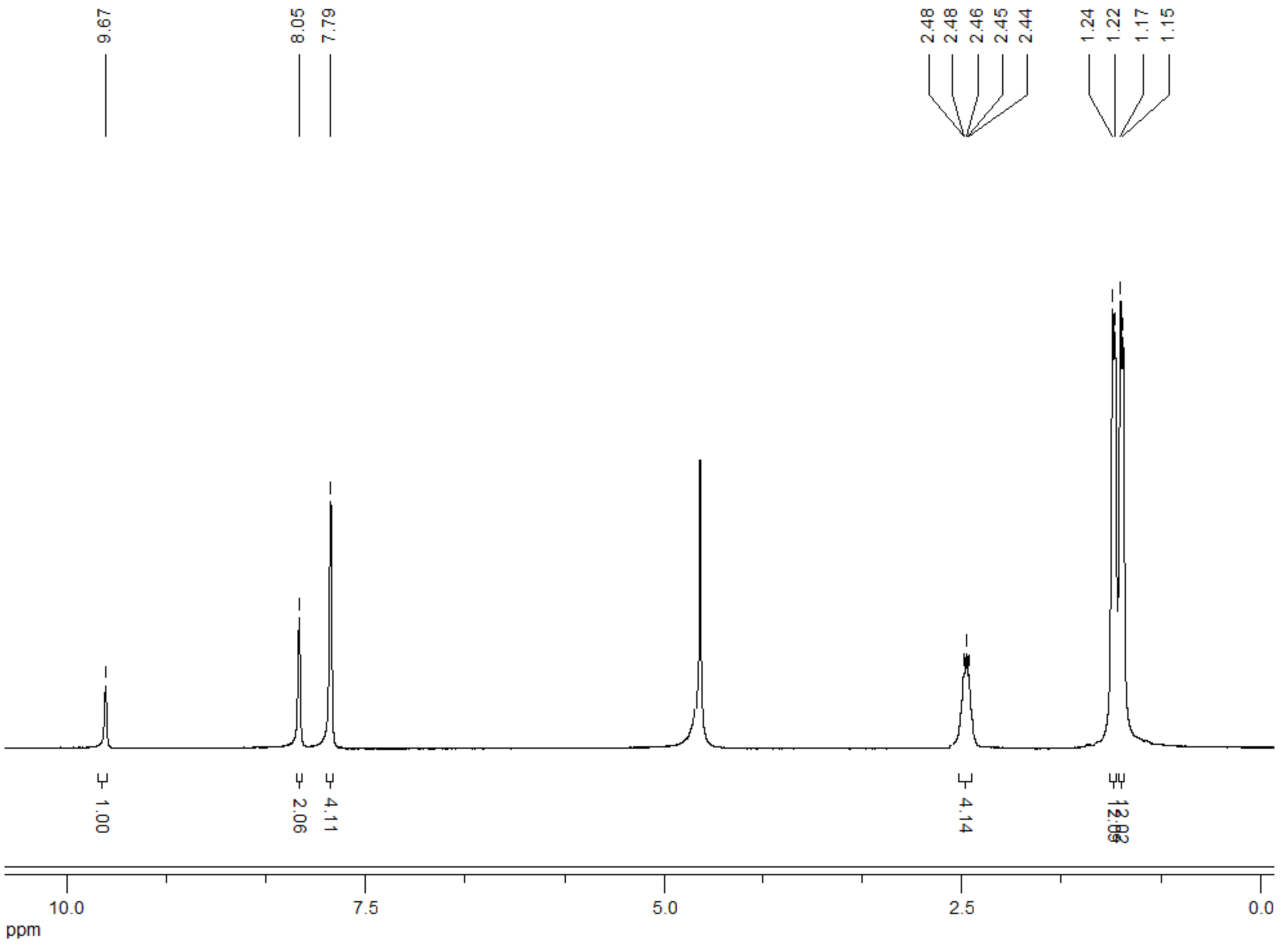

1,3-bis(2,6-diisopropyl-4sodiumsulfonatophenyl)imidazolium (**9**) (^{13}C-NMR, D_2O)

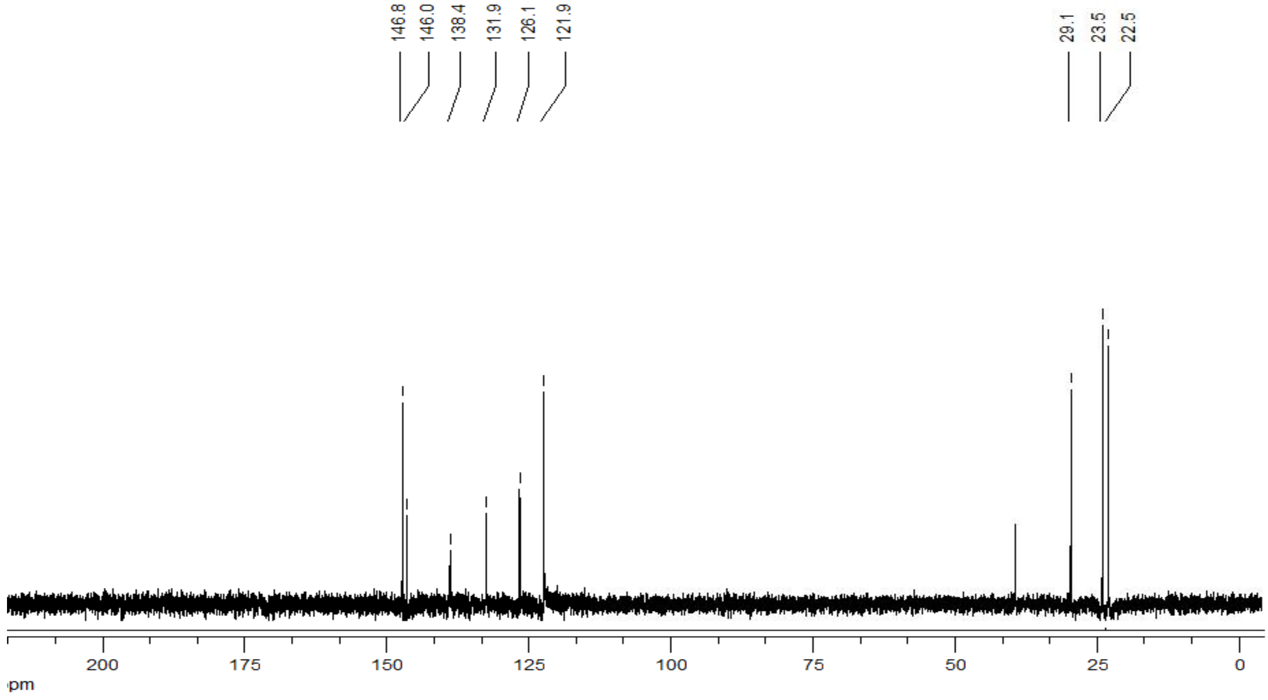

Mesityl imidazol (**10**) (^{1}H-NMR, $CDCl_3$)

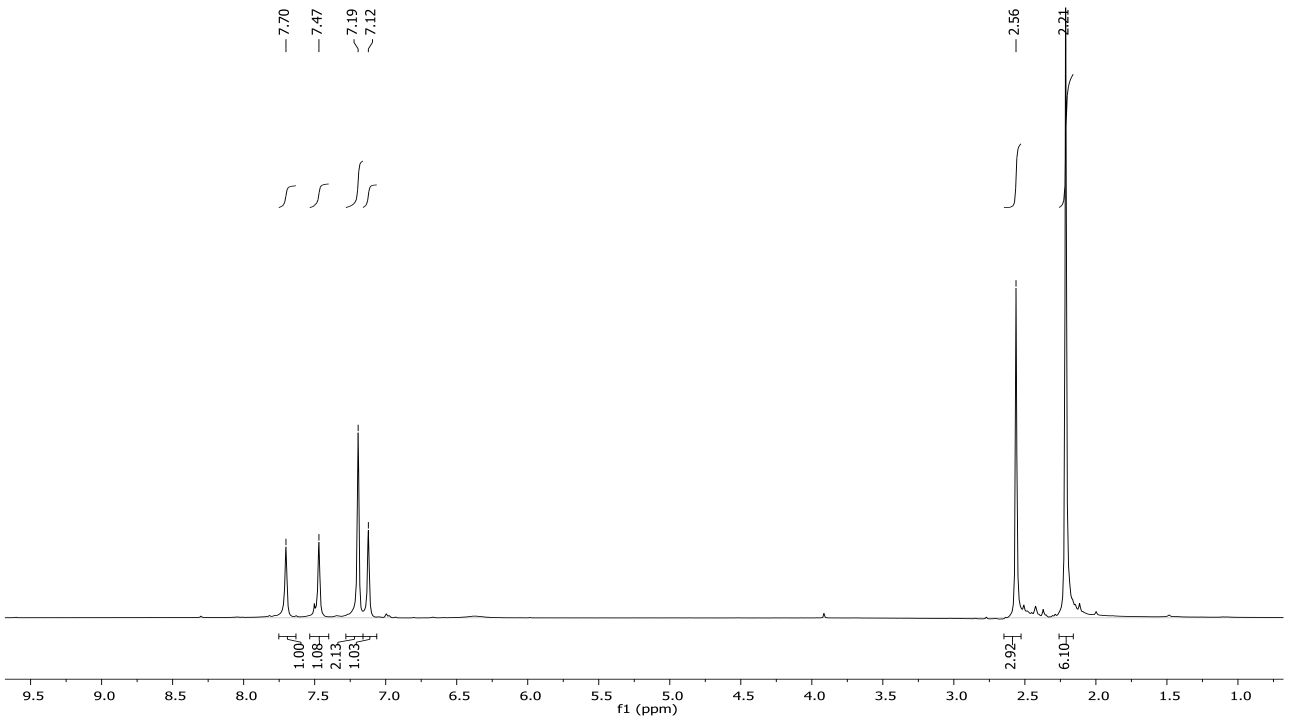

Mesityl imidazol (**10**) (^{13}C-NMR, $CDCl_3$)

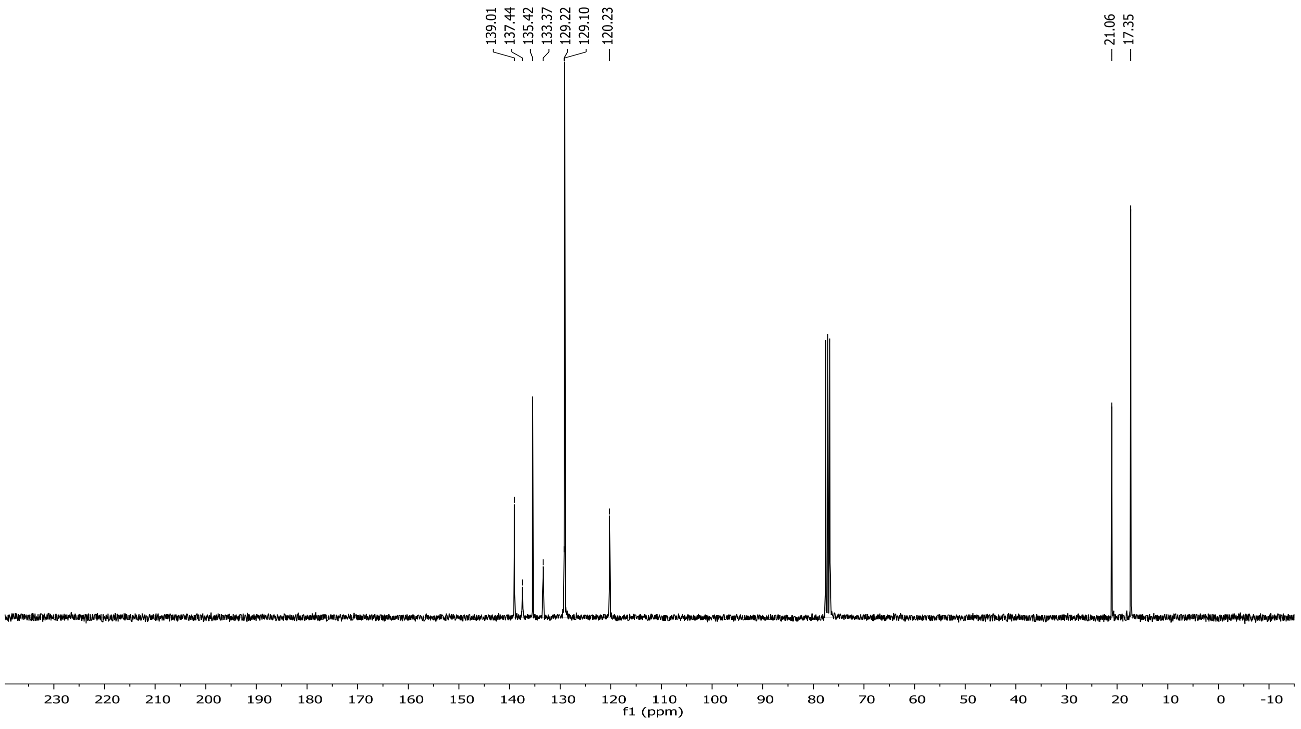

Diisopropyl imidazol (**11**) (^{1}H-NMR, $CDCl_3$)

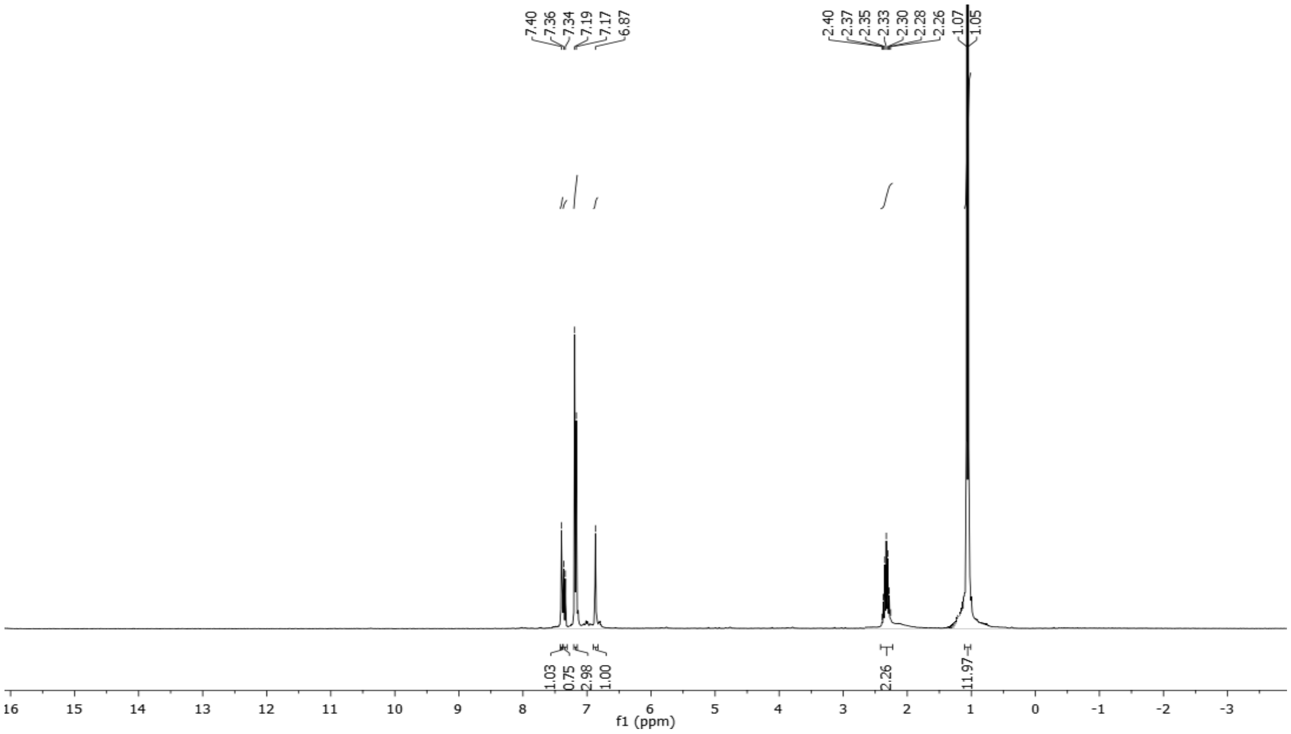

Diisopropyl imidazol (**11**) (^{13}C-NMR, $CDCl_3$)

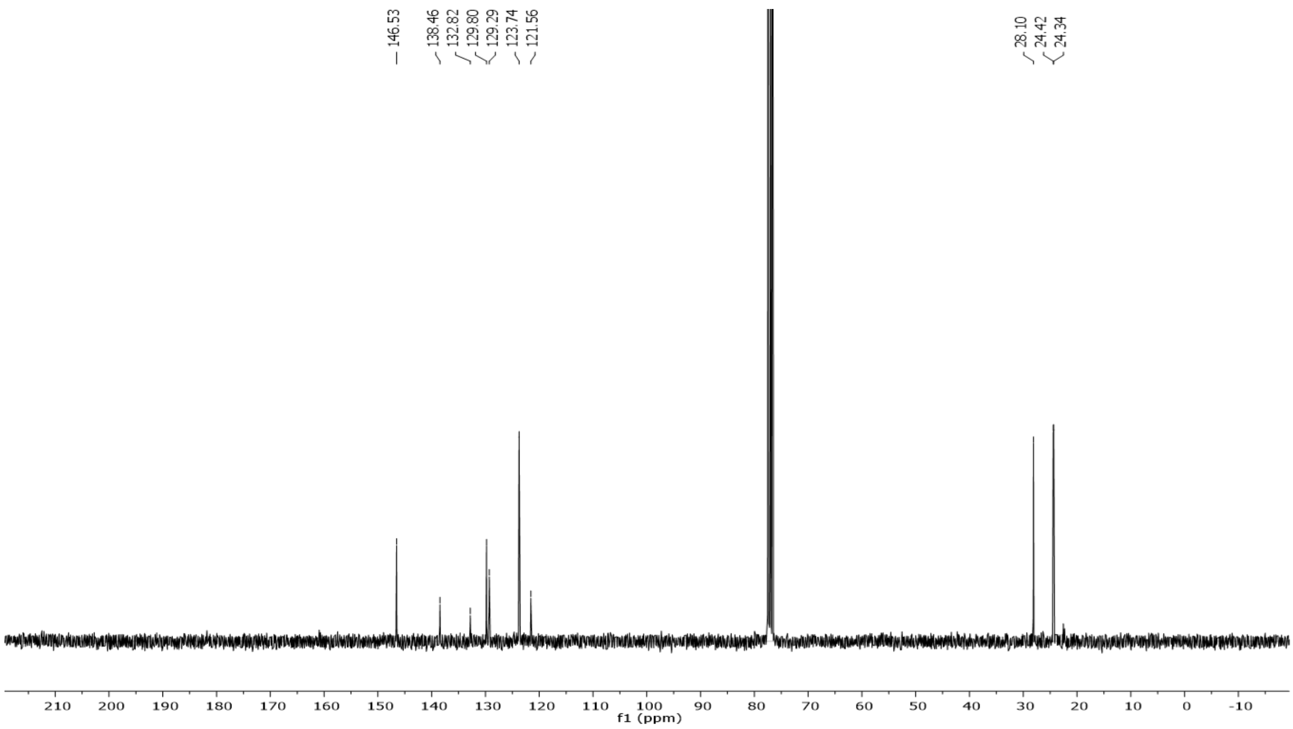

Supplement: S1 Appendix — (PDF) [file pntd.0007021.s009.pdf]
